# Supplementary material for: Elemental Analysis and In Vitro Evaluation of Antibacterial and Antifungal Activities of Usnea barbata (L.) Weber ex F.H. Wigg from Călimani Mountains, Romania
Source: Plants (Basel). 2021 Dec 23;11(1):32. doi: 10.3390/plants11010032 (PMC8747648; doi:10.3390/plants11010032)
Supplement: Supplementary file 1 [file plants-11-00032-s001.zip › Calibration standard solutions preparation and detection limits.pdf]

For calibration standard solutions (Cal.Std.) preparation, the suitable standard solutions are registered in Table S3.

Table S3. ICP-MS Standard solutions

Solution	Concentration	Manufacturer
Arsenic Standard Solution	1000 µg/mL	Perkin Elmer
Lead Standard Solution	1000 µg/mL	Perkin Elmer
Cadmium Standard Solution	1000 µg/mL	Perkin Elmer
Mercury Standard Solution	10 µg/mL	Perkin Elmer
Aluminium Standard Solution	1000 µg/mL	Perkin Elmer
Calcium Standard Solution	1000 µg/mL	Perkin Elmer
Iron Standard Solution	1000 µg/mL	Perkin Elmer
Magnesium Standard Solution	1000 µg/mL	Perkin Elmer
Manganase Standard Solution	1000 µg/mL	Perkin Elmer
Zinc Standard Solution	1000 µg/mL	Perkin Elmer
Silver Standard Solution	1000 µg/mL	Perkin Elmer
Barium Standard Solution	1000 µg/mL	Perkin Elmer
Cobalt Standard Solution	1000 µg/mL	Perkin Elmer
Chromium Standard Solution	1000 µg/mL	Perkin Elmer
Cooper Standard Solution	1000 µg/mL	Perkin Elmer
Lithium Standard Solution	1000 µg/mL	Perkin Elmer
Nickel Standard Solution	1000 µg/mL	Perkin Elmer
Thallium Standard Solution	1000 µg/mL	Perkin Elmer
Vanadium Standard Solution	1000 µg/mL	Perkin Elmer
Molybdenum Standard Solution	1000 µg/mL	Perkin Elmer
Palladium Standard Solution	1000 µg/mL	Perkin Elmer
Platinum Standard Solution	1000 µg/mL	Perkin Elmer
Antimony Standard Solution	1000 µg/mL	Perkin Elmer
NexION Setup Solution (Be, Ce, Fe, In, Li, Mg, Pb, U)	1 µg/L	Perkin Elmer
NexION KED Mode Setup Solution (Be, Ce, Fe, In, Li, Mg, Pb, U)	1 µg/L	Perkin Elmer

Four intermediary stock solutions (Sol. I – Sol. IV) were prepared as follows:

Sol. I (As, Pb, Cd, Hg) 1 mg/L: In a 20 mL volumetric flask were added: 0.2 mL 65% HNO_3, 0.02 mL solution As 1000 mg / L, 0.02 mL solution Pb 1000 mg / L, 0.02 mL solution Cd 1000 mg / L and 2 mL solution Hg 10 mg / L; then, the obtained solution was brought to the mark with ultrapure water.

Sol. II (Ca, Fe, Mg, Mn, Zn) 10 mg/L: In a 50 mL volumetric flask were added: 0.5 mL 65% HNO_3, 0.5 mL solution Ca 1000 mg / L, 0.5 mL solution Fe 1000 mg / L, 0.5 mL solution Mg 1000 mg / L, 0.5 mL solution Mn 1000 mg / L and 0.5 mL solution Zn 1000 mg / L; then, the flask content was brought to the mark with ultrapure water.

Sol. III (Al) 10 mg/L: In a 50 mL volumetric flask were added 0.5 mL 65% HNO_3, 0.5 mL solution Al 1000 mg/L, and ultrapure water up to the mark.

Sol. IV (Ag, Ba, Co, Cr, Cu, Li, Ni, Tl, V, Mo, Pd, Pt, Sb) 1 mg/L: In a 50 mL volumetric flask were added: 0.5 mL 65% HNO_3, 0.05 mL solution Ag 1000 mg / L, 0.05 mL solution Ba 1000 mg / L, 0.05

mL solution Co 1000 mg / L, 0.05 mL solution Cr 1000 mg / L, 0.05 mL solution Cu, 0.05 mL solution Li 1000 mg / L, 0.05 mL solution Ni 1000 mg / L, 0.05 mL solution Tl 1000 mg / L, 0.05 mL solution V 1000 mg / L, 0.05 mL solution Mo 1000 mg / L, 0.05 mL solution Pd 1000 mg / L, 0.05 mL solution Pt 1000 mg / L and 0.05 mL solution Sb 1000 mg / L; then, the obtained solution was brought to the mark with ultrapure water.

The four intermediate stock solutions (Sol. I – Sol. IV) were used for Cal.Std. Solutions (E1-E5) preparation. Thus, in each 25 mL volumetric flask were added 4 mL 65% HNO_3, different volumes (mL) of Sol. I – Sol. IV (as registered in table S4) and ultrapure water up to the mark.

Table S4. Preparation of (E1-E5) calibration standard solutions

Solution	E1	E2	E3	E4	E5
			Volume (mL)		
**65% HNO_3**	4.000	4.000	4.000	4.000	4.000
**Sol. I**	0.025	0.125	0.250	0.375	0.625
**Sol. II**	0.125	0.250	0.500	0.750	1.250
**Sol. III**	0.025	0.125	0.250	0.375	0.500
**Sol. IV**	0.025	0.125	0.250	1.250	2.500

Figure S1 represents the calibration curves for 23 elements in ICP-MS analysis, where:

- Apparent concentration is each (E1-E5) calibration standard solution concentration (μg/L)
- Net intensity is the intensity of the signal received by the ICP detector, corresponding to the number of metal ions striking the detector every second (measured in count per second = CPS) from each (E1-E5) calibration standard solution
- Eqn.=linear equation
- Cor.Coeff.= coefficient of correlation (R^2)
- BEC = background equivalent concentration (μg/L)
- DL=detection limit or limit of detection (LOD, μg/L) (https://resources.perkinelmer.com/labsolutions/resources/docs/WHP_Atomic_Spectroscopy-Effects_on_Accuracy_and_Detection_Limits_013559_01.pdf)

Table S5. Calibration curve range, R^2, LOD, and LOQ (μg/L and μg/g) for each element

Element	m/z	Calibration curve range		R^2	LOD		LOQ estimated value	
		μg/L	μg/g		μg/L	μg/g	μg/L	μg/g
**Ag**	107	1 – 100	0.1 – 10	0.999997	0.058	0.006	1.000	0.100
**Al**	27	10 – 200	1 – 20	0.999872	2.863	0.286	10.00	1.000
**As**	75	1 – 25	0.1 – 2.5	0.999861	0.345	0.035	1.000	0.100
**Ba**	138	1 – 100	0.1 – 10	0.999992	0.067	0.007	1.000	0.100
**Ca**	43	50 – 500	5 – 50	0.996738	4.924	0.492	50.00	5.000
**Cd**	111	1 – 25	0.1 – 2.5	0.999853	0.147	0.015	1.000	0.100
**Co**	59	1 – 100	0.1 – 10	0.999982	0.060	0.006	1.000	0.100
**Cr**	52	1 – 100	0.1 – 10	0.999976	0.090	0.009	1.000	0.100
**Cu**	63	1 – 100	0.1 – 10	0.999995	0.057	0.006	1.000	0.100
**Fe**	57	50 – 500	5 – 50	0.998754	3.392	0.339	50.00	5.000
**Li**	7	1 – 100	0.1 – 10	0.999932	0.198	0.020	1.000	0.100
**Mg**	24	50 – 500	5 – 50	0.999858	2.523	0.252	50.00	5.000
**Mn**	55	50 – 500	5 – 50	0.999801	1.911	0.191	50.00	5.000
**Ni**	60	1 – 100	0.1 – 10	0.999986	0.096	0.010	1.000	0.100
**Pb**	208	1 – 25	0.1 – 2.5	0.999646	0.053	0.005	1.000	0.100
**Tl**	205	1 – 100	0.1 – 10	0.999985	0.025	0.002	1.000	0.100
**V**	51	1 – 100	0.1 – 10	0.999991	0.103	0.010	1.000	0.100
**Zn**	66	50 – 500	5 – 50	0.999791	1.996	0.200	50.00	5.000
**Hg**	202	1 – 25	0.1 – 2.5	0.999751	0.198	0.020	1.000	0.100
**Mo**	98	1 – 100	0.1 – 10	0.999995	0.097	0.010	1.000	0.100
**Pd**	106	1 – 100	0.1 – 10	0.999998	0.064	0.006	1.000	0.100
**Pt**	195	1 – 100	0.1 – 10	0.999998	0.027	0.003	1.000	0.100
**Sb**	121	1 – 100	0.1 – 10	0.999984	0.138	0.014	1.000	0.100

m/z - mass-to-charge ratio; R^2 – correlation coefficient > 0.99; LOD – limit of detection, LOQ – Limit of quantification; Ag – silver, Al – aluminium, As – arsenic, Ba – barium, Ca – calcium, Cd—cadmium, Co—cobalt, Cr—chromium, Cu—copper, Fe—iron, Li—lithium, Mg—magnesium, Mn—manganese, Ni—nickel, Pb – lead, Tl—thallium, Zn—zinc, Hg – mercury, Mo—molybdenum, Pd – palladium, Pt – platinum, Sb—antimony
