# Supplementary material for: Elemental Analysis and In Vitro Evaluation of Antibacterial and Antifungal Activities of Usnea barbata (L.) Weber ex F.H. Wigg from Călimani Mountains, Romania
Source: Plants (Basel). 2021 Dec 23;11(1):32. doi: 10.3390/plants11010032 (PMC8747648; doi:10.3390/plants11010032)
Supplement: Supplementary file 1 [file plants-11-00032-s001.zip › Elemental standard solutions - Certificates of analysis.pdf]

PerkinElmer Pure Plus
Atomic Spectroscopy Standard

ISO 9001 CERTIFIED

Certificate of Analysis

PerkinElmer Number: N9303726 **Lot No:** CL11-127ALY1

Element and Matrix: 1000 µg/mL Aluminum in 2% HNO3

Starting Material: Aluminum Metal

Starting Material Lot No: 01171B

Certification Date: NOV - - 2020

Density: 1.015 g/mL @ 20°C

Expiration Date: MAY 30 2022

Trace Metallic Impurities in the Actual Solution via ICP / ICP-MS Analysis:

Element	µg/L	Element	µg/L	Element	µg/L	Element	µg/L	Element	µg/L
Ag	1	Dy	<0.05	Li	<0.2	Pt	0.05	Tb	<0.03
As	0.7	Er	<0.03	Lu	<0.01	Rb	<0.08	Te	0.4
Au	0.1	Eu	<0.02	Mg	0.6	Re	<0.03	Th	<0.02
B	1	Fe	0.2	Mn	0.6	Rh	1	Ti	<0.2
Ba	0.3	Ga	<0.3	Mo	<0.5	Ru	<0.2	Tl	<0.2
Be	<0.8	Gd	<0.09	Na	4	Sb	<0.09	Tm	<0.01
Bi	<0.3	Ge	<0.5	Nb	<0.1	Sc	<0.2	U	<0.01
Ca	10	Hf	<0.03	Nd	0.05	Se	<8	V	<0.05
Cd	<0.2	Hg	0.4	Ni	<0.6	Si	100	W	0.3
Ce	<0.04	Ho	<0.02	P	<200	Sm	<0.04	Y	0.07
Co	0.2	In	<0.04	Pb	<0.03	Sn	0.2	Yb	<0.03
Cr	<0.5	Ir	<0.3	Pd	0.5	Sr	0.04	Zn	<0.7
Cs	<0.09	K	1	Pr	<0.02	Ta	<0.5	Zr	<0.2
Cu	2	La	<0.03						

Traceability Documentation for Solution Standard:

Certified Value: 1002 µg/mL ±5 µg/mL (refer to side 2)

Certified Value is Traceable to: NIST SRM #3101a

*** Classical Wet Assay:** 1003 µg/mL

Method: Precipitation using 8 Hydroxy Quinoline. Filter, dry and weigh as Al(C9H6NO)3.

*** Instrumental Analysis using OPTIMA 7300 DV ICP Spectrometer:** 1000 µg/mL
via NIST SRM #3101a

We guarantee that our PerkinElmer Pure Plus Atomic Spectroscopy Standards are stable and accurate to ±0.5% of certified concentration until the expiration date, provided the standards are kept tightly capped and stored under normal laboratory conditions. This value is the sum of cumulative errors associated with the analytical determinations, pipetting, and diluting to final volume. For these solutions we use high purity acids, ASTM Type 1 water (18 megohm double deionized), and leached, triple-rinsed bottles. All glassware used is class A.

Certifying Officer: Y. Parikh

PerkinElmer®

PerkinElmer, Inc.

U.S.A. Tel: 1-203-925-4600
U.S.A. Toll Free: 1-800-762-4000

Visit www.perkinelmer.com/lasoffices for a complete listing of our global offices.

PerkinElmer®

PerkinElmer Pure Plus

PerkinElmer Number: N9303727

Element and Matrix: 1000 µg/mL Arsenic in 2% HNO3

Starting Material: Arsenic Metal

Starting Material Lot No: 12171B

Density: 1.010 g/mL @ 20°C

Lot No: CL12-08ASY1

Certification Date: DEC - - 2020

Expiration Date: JUN 3 0 2022

Trace Metallic Impurities in the Actual Solution via ICP / ICP-MS Analysis:

Element	µg/L	Element	µg/L	Element	µg/L	Element	µg/L	Element	µg/L
Ag	<0.4	Dy	<0.01	Li	<0.2	Pt	<0.04	Tb	<0.01
Al	0.6	Er	<0.01	Lu	<0.01	Rb	<0.03	Te	<0.5
Au	<0.03	Eu	<0.01	Mg	0.2	Re	<0.08	Th	<0.01
B	<2	Fe	0.7	Mn	0.1	Rh	<0.07	Ti	<0.2
Ba	<0.06	Ga	<0.1	Mo	<0.1	Ru	<0.08	Tl	<0.2
Be	<0.05	Gd	<0.03	Na	2	Sb	<0.03	Tm	<0.01
Bi	<0.02	Ge	<0.4	Nb	0.2	Sc	<0.05	U	<0.03
Ca	3	Hf	<0.03	Nd	<0.01	Se	<10	V	0.05
Cd	<0.1	Hg	<0.3	Ni	<0.3	Si	<100	W	<0.2
Ce	0.1	Ho	<0.01	P	<300	Sm	<0.01	Y	<0.03
Co	<0.2	In	2	Pb	<0.3	Sn	1	Yb	<0.01
Cr	<0.4	Ir	<0.09	Pd	<0.07	Sr	<0.03	Zn	0.6
Cs	<0.02	K	4	Pr	<0.01	Ta	<0.2	Zr	<0.2
Cu	<0.3	La	0.01						

Traceability Documentation for Solution Standard:

Certified Value: 1000 µg/mL ±5 µg/mL (refer to side 2)

Certified Value is Traceable to: NIST SRM #3103a

*** Classical Wet Assay:** 1000 µg/mL

Method: Precipitation using Silver Nitrate. Filter, dry, and weigh as Ag3AsO4.

***Instrument Analysis using ICP Spectrometer:** 1000 µg/mL
via NIST SRM #3103a

We guarantee that our PerkinElmer Pure Plus Atomic Spectroscopy Standards are stable and accurate to ±0.5% of certified concentration until the expiration date, provided the standards are kept tightly capped and stored under normal laboratory conditions. This value is the sum of cumulative errors associated with the analytical determinations, pipetting, and diluting to final volume. For these solutions we use high purity acids, ASTM Type 1 water (18 megohm double deionized), and leached, triple-rinsed bottles. All glassware used is class A.

Certifying Officer: Y. Parikh
Yogesh Parikh, Senior Spectroscopist

PerkinElmer®

PerkinElmer Pure

PerkinElmer Number: N9300181

Element and Matrix: 1000 µg/mL Barium in 2% HNO3

Starting Material: Barium Nitrate

Starting Material Lot No: 05161C

Density: 1.011 g/mL @ 20°C

Lot No: 25-31BAY1

Certification Date: MAR -- 2021

Expiration Date: SEP 30 2022

Trace Metallic Impurities in the Actual Solution via ICP / ICP-MS Analysis:

Element	µg/mL	Element	µg/mL	Element	µg/mL	Element	µg/mL	Element	µg/mL
Ag	<0.001	Dy	<0.001	Li	<0.001	Pt	<0.001	Tb	<0.001
Al	<0.004	Er	<0.001	Lu	<0.001	Rb	<0.001	Te	<0.001
As	<0.001	Eu	<0.01	Mg	<0.001	Re	<0.001	Th	<0.001
Au	<0.001	Fe	<0.004	Mn	<0.001	Rh	<0.001	Ti	<0.001
B	<0.001	Ga	<0.001	Mo	<0.001	Ru	<0.001	Tl	<0.001
Be	<0.001	Gd	0.001	Na	0.005	Sb	<0.001	Tm	<0.001
Bi	<0.003	Ge	<0.002	Nb	<0.001	Sc	<0.001	U	<0.001
Ca	0.01	Hf	<0.001	Nd	<0.001	Se	<0.02	V	<0.001
Cd	<0.001	Hg	<0.001	Ni	<0.001	Si	<0.1	W	<0.001
Ce	<0.001	Ho	<0.001	P	<0.5	Sm	0.001	Y	0.007
Co	<0.001	In	<0.001	Pb	<0.001	Sn	<0.001	Yb	<0.001
Cr	<0.001	Ir	<0.001	Pd	<0.001	Sr	0.003	Zn	0.006
Cs	<0.001	K	0.002	Pr	<0.001	Ta	<0.001	Zr	<0.001
Cu	<0.001	La	0.009						

Traceability Documentation for Solution Standard:

Certified Value: 999 µg/mL ±5 µg/mL (refer to side 2)

Certified Value is Traceable to: NIST SRM #3104a

*** Classical Wet Assay:** 997 µg/mL

Method: Precipitation using Sulfuric Acid. Filter, ignite, and weigh as BaSO4.

***Instrument Analysis using ICP Spectrometer:** 1000 µg/mL
via NIST SRM #3104a

We guarantee that our PerkinElmer Pure Atomic Spectroscopy Standards are stable and accurate to ±0.5% of certified concentration until the expiration date, provided the standards are kept tightly capped and stored under normal laboratory conditions. This value is the sum of cumulative errors associated with the analytical determinations, pipetting, and diluting to final volume. For these solutions we use high purity acids, ASTM Type 1 water (18 megohm double deionized), and leached, triple-rinsed bottles. All glassware used is class A.

Certifying Officer: Y. Parikh
Yogesh Parikh, Senior Spectroscopist

PerkinElmer Pure Plus

Atomic Spectroscopy Standard

ISO 9001 CERTIFIED

Certificate of Analysis

PerkinElmer Number: N9303734 **Lot No:** CL11-132CDY1

Element and Matrix: 1000 µg/mL Cadmium in 2% HNO3

Starting Material: Cadmium Oxide

Starting Material Lot No: 11151A

Certification Date: AUG -- 2020

Density: 1.010 g/mL @ 20°C

Expiration Date: FEB 28 2022

Trace Metallic Impurities in the Actual Solution via ICP / ICP-MS Analysis:

Element	µg/L	Element	µg/L	Element	µg/L	Element	µg/L	Element	µg/L
Ag	<0.1	Dy	<0.04	Li	<0.3	Pt	<0.2	Tb	0.01
Al	2	Er	<0.05	Lu	<0.01	Rb	<0.09	Te	<2
As	<0.6	Eu	2	Mg	0.3	Re	<0.03	Th	<0.02
Au	<0.1	Fe	3	Mn	<1	Rh	<0.8	Ti	<0.09
B	0.9	Ga	<0.2	Mo	<0.2	Ru	<0.2	Tl	<0.2
Ba	<0.5	Gd	<0.1	Na	3	Sb	<0.04	Tm	<0.01
Be	<0.2	Ge	<1	Nb	<0.5	Sc	<0.2	U	<0.01
Bi	<0.01	Hf	<0.04	Nd	<0.05	Se	<0.5	V	<0.3
Ca	8	Hg	<0.3	Ni	<0.7	Si	<100	W	<0.3
Ce	<0.07	Ho	<0.01	P	<100	Sm	<0.01	Y	<0.05
Co	<0.1	In	0.8	Pb	<0.03	Sn	<0.2	Yb	<0.05
Cr	0.6	Ir	<0.2	Pd	<0.3	Sr	<0.04	Zn	3
Cs	<0.1	K	5	Pr	<0.05	Ta	<0.08	Zr	<0.5
Cu	<0.3	La	<0.07						

Traceability Documentation for Solution Standard:

Certified Value: 1000 µg/mL ±5 µg/mL (refer to side 2)

Certified Value is Traceable to: NIST SRM #3108

*** Classical Wet Assay:** 998 µg/mL

Method: EDTA titration using Xylenol Orange as indicator. EDTA standardized against Pb(NO3)2 NIST SRM #928.

*** Instrumental Analysis using OPTIMA 7300 DV ICP Spectrometer:** 1001 µg/mL **via** NIST SRM #3108

We guarantee that our PerkinElmer Pure Plus Atomic Spectroscopy Standards are stable and accurate to ±0.5% of certified concentration until the expiration date, provided the standards are kept tightly capped and stored under normal laboratory conditions. This value is the sum of cumulative errors associated with the analytical determinations, pipetting, and diluting to final volume. For these solutions we use high purity acids, ASTM Type 1 water (18 megohm double deionized), and leached, triple-rinsed bottles. All glassware used is class A.

Certifying Officer: Y. Parikh

PerkinElmer®

PerkinElmer, Inc.

U.S.A. Tel: 1-203-925-4600

U.S.A. Toll Free: 1-800-762-4000

Visit www.perkinelmer.com/lasoffices for a complete listing of our global offices.

PerkinElmer

PerkinElmer Pure Plus

PerkinElmer Number: N9303733

Element and Matrix: 1000 µg/mL Calcium in 2% HNO3

Starting Material: Calcium Carbonate

Starting Material Lot No: 10141C

Density: 1.011 g/mL @ 20°C

Lot No: CL11-198CAY1

Certification Date: JAN - - 2021

Expiration Date: JUL 3 0 2022

Trace Metallic Impurities in the Actual Solution via ICP / ICP-MS Analysis:

Element	µg/L	Element	µg/L	Element	µg/L	Element	µg/L	Element	µg/L
Ag	<0.2	Dy	<0.01	Li	0.3	Pt	<0.08	Tb	<0.01
Al	2	Er	<0.01	Lu	<0.02	Rb	<0.02	Te	<2
As	0.6	Eu	<0.02	Mg	0.4	Re	<0.02	Th	<0.04
Au	<0.3	Fe	<2	Mn	<0.3	Rh	<0.04	Ti	2
B	<2	Ga	<0.06	Mo	<0.2	Ru	<0.6	Tl	<0.9
Ba	1	Gd	<0.01	Na	2	Sb	<0.3	Tm	<0.01
Be	<0.09	Ge	<0.5	Nb	<0.04	Sc	<0.4	U	<0.01
Bi	<0.01	Hf	<0.01	Nd	<0.01	Se	<20	V	<0.08
Cd	<0.09	Hg	<0.7	Ni	<0.7	Si	<100	W	<0.6
Ce	0.4	Ho	<0.01	P	<200	Sm	<0.01	Y	<0.02
Co	<0.2	In	<0.04	Pb	0.05	Sn	<0.1	Yb	<0.01
Cr	<0.8	Ir	<0.1	Pd	<0.4	Sr	10	Zn	<2
Cs	0.1	K	2	Pr	<0.01	Ta	<0.08	Zr	<0.2
Cu	<0.3	La	0.2						

Traceability Documentation for Solution Standard:

Certified Value: 1000 µg/mL ±5 µg/mL (refer to side 2)

Certified Value is Traceable to: NIST SRM #3109a

*** Classical Wet Assay:** 999 µg/mL

Method: EDTA titration using Hydroxy Naphthol Blue as indicator. EDTA standardized against Pb(NO3)2 NIST SRM #928

***Instrument Analysis using ICP Spectrometer:** 1000 µg/mL
via NIST SRM #3109a

We guarantee that our PerkinElmer Pure Plus Atomic Spectroscopy Standards are stable and accurate to ±0.5% of certified concentration until the expiration date, provided the standards are kept tightly capped and stored under normal laboratory conditions. This value is the sum of cumulative errors associated with the analytical determinations, pipetting, and diluting to final volume. For these solutions we use high purity acids, ASTM Type 1 water (18 megohm double deionized), and leached, triple-rinsed bottles. All glassware used is class A.

Certifying Officer: Y. Parikh
Yogesh Parikh, Senior Spectroscopist

PerkinElmer®

PerkinElmer Pure Plus

**PerkinElmer Number:**	N9303736	**Lot No:**	CL11-141CRY1
**Element and Matrix:**	1000 µg/mL Chromium in 2% HNO3	**Certification Date:**	NOV - - 2020
**Starting Material:**	Chromium(III) Nitrate Nonahydrate	**Expiration Date:**	MAY 30 2022
**Starting Material Lot No:**	03161L		
**Density:**	1.013 g/mL @ 20°C		

Trace Metallic Impurities in the Actual Solution via ICP / ICP-MS Analysis:

Element	µg/L	Element	µg/L	Element	µg/L	Element	µg/L	Element	µg/L
Ag	<0.6	Dy	<0.02	Li	<0.3	Pt	<0.03	Tb	<0.01
Al	4	Er	<0.01	Lu	<0.01	Rb	10	Te	<0.7
As	1	Eu	<0.01	Mg	10	Re	<0.07	Th	<0.01
Au	<0.4	Fe	30	Mn	0.5	Rh	2	Ti	1
B	<2	Ga	2	Mo	<2	Ru	20	Tl	<0.2
Ba	0.1	Gd	<0.03	Na	7	Sb	<0.7	Tm	<0.01
Be	0.07	Ge	<0.6	Nb	2	Sc	<0.7	U	<0.01
Bi	<0.05	Hf	0.04	Nd	<0.01	Se	<20	V	2
Ca	50	Hg	<0.07	Ni	0.5	Si	<100	W	<0.4
Cd	<0.2	Ho	<0.01	P	<400	Sm	<0.01	Y	<0.02
Ce	0.3	In	0.2	Pb	0.1	Sn	<0.8	Yb	0.04
Co	<0.2	Ir	<0.2	Pd	<0.2	Sr	<0.4	Zn	20
Cs	<0.03	K	4	Pr	0.01	Ta	<0.1	Zr	0.3
Cu	4	La	<0.04						

Traceability Documentation for Solution Standard:

**Certified Value:**	1003 µg/mL ±5 µg/mL (refer to side 2)
**Certified Value is Traceable to:**	NIST SRM #3112a
*** Classical Wet Assay:**	1004 µg/mL
**Method:**	Precipitation using Ammonium Hydroxide. Filter, ignite, and weigh as Cr2O3.

***Instrument Analysis using ICP Spectrometer:** 1001 µg/mL
via NIST SRM #3112a

We guarantee that our PerkinElmer Pure Plus Atomic Spectroscopy Standards are stable and accurate to ±0.5% of certified concentration until the expiration date, provided the standards are kept tightly capped and stored under normal laboratory conditions. This value is the sum of cumulative errors associated with the analytical determinations, pipetting, and diluting to final volume. For these solutions we use high purity acids, ASTM Type 1 water (18 megohm double deionized), and leached, triple-rinsed bottles. All glassware used is class A.

Certifying Officer: Y. Parikh
Yogesh Parikh, Senior Spectroscopist

PerkinElmer®

PerkinElmer Pure Plus

PerkinElmer Number: N9303735

Element and Matrix: 1000 µg/mL Cobalt in 2% HNO3

Starting Material: Cobalt Metal (powder)

Starting Material Lot No: 10181G

Density: 1.011 g/mL @ 20°C

Lot No: CL11-168COY1

Certification Date: OCT -- 2020

Expiration Date: APR 30 2022

Trace Metallic Impurities in the Actual Solution via ICP / ICP-MS Analysis:

Element	µg/L	Element	µg/L	Element	µg/L	Element	µg/L	Element	µg/L
Ag	<0.5	Dy	<0.01	Li	<0.5	Pt	<0.2	Tb	<0.01
Al	1	Er	<0.01	Lu	<0.01	Rb	<0.2	Te	<0.5
As	<20	Eu	<0.01	Mg	0.2	Re	<0.05	Th	<0.08
Au	<0.2	Fe	<0.8	Mn	<0.4	Rh	<0.09	Ti	0.1
B	<1	Ga	<0.09	Mo	<0.1	Ru	<0.2	Tl	<0.8
Ba	<0.01	Gd	<0.03	Na	<0.3	Sb	<0.06	Tm	<0.01
Be	0.1	Ge	0.4	Nb	<0.3	Sc	<0.08	U	<0.01
Bi	<0.6	Hf	<0.07	Nd	<0.01	Se	<4	V	<0.1
Ca	8	Hg	<0.3	Ni	2	Si	<200	W	<0.5
Cd	<0.3	Ho	<0.01	P	<400	Sm	<0.01	Y	<0.02
Ce	<0.01	In	<0.06	Pb	<0.3	Sn	<0.1	Yb	<0.01
Cr	0.2	Ir	<0.2	Pd	<0.3	Sr	<0.04	Zn	1
Cs	<0.2	K	3	Pr	<0.01	Ta	<0.7	Zr	<0.3
Cu	<0.2	La	<0.02						

Traceability Documentation for Solution Standard:

Certified Value: 1001 µg/mL ±5 µg/mL (refer to side 2)

Certified Value is Traceable to: NIST SRM #3113

*** Classical Wet Assay:** 1003 µg/mL

Method: EDTA titration using Xylenol Orange as indicator. EDTA standardized against Pb(NO3)2 NIST SRM # 928.

***Instrument Analysis using ICP Spectrometer:** 999 µg/mL
via NIST SRM #3113

We guarantee that our PerkinElmer Pure Plus Atomic Spectroscopy Standards are stable and accurate to ±0.5% of certified concentration until the expiration date, provided the standards are kept tightly capped and stored under normal laboratory conditions. This value is the sum of cumulative errors associated with the analytical determinations, pipetting, and diluting to final volume. For these solutions we use high purity acids, ASTM Type 1 water (18 megohm double deionized), and leached, triple-rinsed bottles. All glassware used is class A.

Certifying Officer: Y. Parikh
Yogesh Parikh, Senior Spectroscopist

PerkinElmer Pure Plus

Atomic Spectroscopy Standard

ISO 9001 CERTIFIED

Certificate of Analysis

PerkinElmer Number: N9303737 **Lot No:** CL11-136CUY1

Element and Matrix: 1000 µg/mL Copper in 2% HNO3

Starting Material: Copper Metal

Starting Material Lot No: 11141I

Certification Date: DEC -- 2020

Density: 1.011 g/mL @ 20°C

Expiration Date: JUN 30 2022

Trace Metallic Impurities in the Actual Solution via ICP / ICP-MS Analysis:

Element	µg/L	Element	µg/L	Element	µg/L	Element	µg/L	Element	µg/L
Ag	<0.2	Dy	<0.01	Li	<0.2	Pt	<0.04	Tb	<0.01
Al	0.5	Er	<0.01	Lu	<0.01	Rb	<0.07	Te	<0.6
As	<0.3	Eu	<0.01	Mg	<0.3	Re	<0.01	Th	<0.03
Au	<0.3	Fe	<0.9	Mn	<0.6	Rh	40	Ti	<0.3
B	<0.8	Ga	<0.06	Mo	<0.2	Ru	0.7	Tl	<1
Ba	<0.08	Gd	<0.01	Na	<1	Sb	<0.2	Tm	<0.01
Be	<0.5	Ge	<0.6	Nb	<0.05	Sc	<0.2	U	<0.01
Bi	<0.02	Hf	<0.01	Nd	<0.01	Se	<10	V	<0.1
Ca	6	Hg	<4	Ni	<0.2	Si	<100	W	<0.1
Cd	<0.1	Ho	<0.01	P	<100	Sm	<0.01	Y	<0.04
Ce	0.03	In	<0.02	Pb	<0.03	Sn	<0.2	Yb	<0.01
Co	<0.3	Ir	<3	Pd	<0.7	Sr	<0.05	Zn	4
Cr	<1	K	2	Pr	<0.01	Ta	<0.1	Zr	<0.2
Cs	<0.05	La	<0.01						

Traceability Documentation for Solution Standard:

Certified Value: 1002 µg/mL ±5 µg/mL (refer to side 2)

Certified Value is Traceable to: NIST SRM #3114

*** Classical Wet Assay:** 1002 µg/mL

Method: EDTA titration using PAN as indicator. EDTA standardized against Pb(NO3)2 NIST SRM #9
28.

*** Instrumental Analysis using OPTIMA 7300 DV ICP Spectrometer:** 1001 µg/mL
via NIST SRM #3114

We guarantee that our PerkinElmer Pure Plus Atomic Spectroscopy Standards are stable and accurate to ±0.5% of certified concentration until the expiration date, provided the standards are kept tightly capped and stored under normal laboratory conditions. This value is the sum of cumulative errors associated with the analytical determinations, pipetting, and diluting to final volume. For these solutions we use high purity acids, ASTM Type 1 water (18 megohm double deionized), and leached, triple-rinsed bottles. All glassware used is class A.

Certifying Officer: Y. Parikh

PerkinElmer®

PerkinElmer, Inc.
U.S.A. Tel: **1-203-925-4600**
U.S.A. Toll Free: **1-800-762-4000**

PerkinElmer®

PerkinElmer Pure Plus

PerkinElmer Number: N9303738

Element and Matrix: 1000 µg/mL Iron in 2% HNO3

Starting Material: Iron Metal

Starting Material Lot No: 10141F

Density: 1.013 g/mL @ 20°C

Lot No: CL12-36FEY1

Certification Date: MAY - - 2021

Expiration Date: NOV 30 2022

Trace Metallic Impurities in the Actual Solution via ICP / ICP-MS Analysis:

Element	µg/L	Element	µg/L	Element	µg/L	Element	µg/L	Element	µg/L
Ag	<0.2	Cu	2	Li	<0.1	Pt	<0.02	Tb	<0.01
Al	0.6	Dy	<0.01	Lu	<0.01	Rb	0.04	Te	<0.1
As	0.5	Er	<0.01	Mg	0.4	Re	<0.04	Th	<0.01
Au	<0.2	Eu	<0.01	Mn	9	Rh	<0.1	Ti	<0.2
B	<0.1	Ga	0.1	Mo	0.4	Ru	0.06	Tl	<0.09
Ba	<0.07	Gd	<0.01	Na	1	Sb	1	Tm	<0.01
Be	<0.3	Ge	<0.3	Nb	<0.02	Sc	<0.07	U	<0.01
Bi	<0.01	Hf	<0.01	Nd	<0.03	Se	<0.3	V	<0.3
Ca	8	Hg	<0.2	Ni	2	Si	<100	W	0.3
Cd	<0.04	Ho	<0.01	P	<200	Sm	<0.01	Y	<0.09
Ce	<0.01	In	<0.03	Pb	0.02	Sn	<0.2	Yb	<0.01
Co	3	Ir	<0.04	Pd	<0.2	Sr	<0.06	Zn	2
Cr	1	K	1	Pr	<0.01	Ta	<0.2	Zr	<0.2
Cs	<0.04	La	<0.01						

Traceability Documentation for Solution Standard:

Certified Value: 1000 µg/mL ±5 µg/mL (refer to side 2)

Certified Value is Traceable to: NIST SRM #3126a

*** Classical Wet Assay:** 1001 µg/mL

Method: Precipitation using Ammonium Hydroxide. Filter, ignite, and weigh as Fe_2O_3.

***Instrument Analysis using ICP Spectrometer:** 999 µg/mL
via NIST SRM #3126a

We guarantee that our PerkinElmer Pure Plus Atomic Spectroscopy Standards are stable and accurate to ±0.5% of certified concentration until the expiration date, provided the standards are kept tightly capped and stored under normal laboratory conditions. This value is the sum of cumulative errors associated with the analytical determinations, pipetting, and diluting to final volume. For these solutions we use high purity acids, ASTM Type 1 water (18 megohm double deionized), and leached, triple-rinsed bottles. All glassware used is class A.

Certifying Officer: Y. Parikh

Yogesh Parikh, Senior Spectroscopist

PerkinElmer

PerkinElmer Pure Plus

**PerkinElmer Number:**	N9303748	**Lot No:**	CL12-64PBY1
**Element and Matrix:**	1000 µg/mL Lead in 2% HNO3	**Certification Date:**	DEC - - 2020
**Starting Material:**	Lead(II) Oxide	**Expiration Date:**	JUN 30 2022
**Starting Material Lot No:**	01851R		
**Density:**	1.010 g/mL @ 20°C		

Trace Metallic Impurities in the Actual Solution via ICP / ICP-MS Analysis:

Element	µg/L	Element	µg/L	Element	µg/L	Element	µg/L	Element	µg/L
Ag	1	Cu	<0.6	La	<0.04	Pt	<0.3	Tb	<0.01
Al	0.7	Dy	<0.01	Li	<0.6	Rb	<0.07	Te	<0.2
As	<0.9	Er	<0.01	Lu	<0.01	Re	<0.03	Th	<0.01
Au	<0.3	Eu	<0.01	Mg	1	Rh	5	Ti	<0.4
B	<0.8	Fe	3	Mn	<0.9	Ru	<0.3	Tl	0.3
Ba	0.07	Ga	<0.03	Mo	<0.2	Sb	<0.09	Tm	<0.01
Be	<2	Gd	<0.04	Na	4	Sc	<0.4	U	<0.01
Bi	10	Ge	<0.4	Nb	<0.3	Se	<8	V	<0.2
Ca	8	Hf	<0.01	Nd	<0.04	Si	<100	W	<0.6
Cd	<0.2	Hg	<0.2	Ni	<0.5	Sm	<0.01	Y	<0.07
Ce	<0.02	Ho	<0.01	P	<400	Sn	<0.07	Yb	<0.01
Co	<0.2	In	<0.04	Pd	<0.2	Sr	<0.1	Zn	0.7
Cr	<0.2	Ir	<0.04	Pr	<0.01	Ta	<0.08	Zr	<0.3
Cs	<0.03	K	4						

Traceability Documentation for Solution Standard:

Certified Value: 1000 µg/mL ±5 µg/mL (refer to side 2)

Certified Value is Traceable to: NIST SRM #3128

*** Classical Wet Assay:** 1001 µg/mL

Method: EDTA titration using Xylenol Orange as indicator. EDTA standardized against Pb(NO3)2 NIST SRM #928

***Instrument Analysis using ICP Spectrometer:** 999 µg/mL
via NIST SRM #3128

We guarantee that our PerkinElmer Pure Plus Atomic Spectroscopy Standards are stable and accurate to ±0.5% of certified concentration until the expiration date, provided the standards are kept tightly capped and stored under normal laboratory conditions. This value is the sum of cumulative errors associated with the analytical determinations, pipetting, and diluting to final volume. For these solutions we use high purity acids, ASTM Type 1 water (18 megohm double deionized), and leached, triple-rinsed bottles. All glassware used is class A.

Certifying Officer: Y. Parikh
Yogesh Parikh, Senior Spectroscopist

PerkinElmer®

PerkinElmer TruQ

**PerkinElmer Number:**	N9303781	**Lot No:**	25-148LIY1
**Element and Matrix:**	1000 µg/mL Lithium in 2% HNO_3	**Certification Date:**	MAY - - 2021
**Starting Material:**	Lithium Carbonate	**Expiration Date:**	NOV 3 0 2022
**Starting Material Lot No:**	11151C		
**Density:**	1.015 g/mL @ 20°C		

Trace Metallic Impurities in the Actual Solution via ICP / ICP-MS Analysis:

Element	µg/mL	Element	µg/mL	Element	µg/mL	Element	µg/mL	Element	µg/mL
Ag	<0.001	Cu	<0.002	La	<0.001	Pt	<0.001	Tb	<0.001
Al	0.003	Dy	<0.001	Lu	<0.001	Rb	<0.001	Te	<0.001
As	<0.001	Er	<0.001	Mg	<0.001	Re	<0.001	Th	<0.001
Au	<0.001	Eu	<0.001	Mn	<0.001	Rh	<0.001	Ti	<0.001
B	<0.02	Fe	<0.004	Mo	<0.001	Ru	<0.001	Tl	<0.001
Ba	0.006	Ga	<0.001	Na	0.02	Sb	<0.001	Tm	<0.001
Be	<0.001	Gd	<0.001	Nb	<0.001	Sc	<0.001	U	<0.001
Bi	<0.001	Ge	<0.002	Nd	<0.001	Se	<0.03	V	<0.001
Ca	0.01	Hf	<0.001	Ni	<0.001	Si	<0.4	W	<0.001
Cd	<0.001	Hg	<0.001	P	<0.5	Sm	<0.001	Y	<0.001
Ce	<0.001	Ho	<0.001	Pb	<0.001	Sn	<0.001	Yb	<0.001
Co	<0.001	In	<0.001	Pd	<0.001	Sr	0.002	Zn	0.01
Cr	<0.001	Ir	<0.001	Pr	<0.001	Ta	<0.001	Zr	<0.001
Cs	<0.001	K	0.003						

Traceability Documentation for Solution Standard:

**Certified Value:**	1000 µg/mL ±5 µg/mL (refer to side 2)
**Certified Value is Traceable to:**	NIST SRM #3129a
*** Classical Wet Assay:**	1001 µg/mL
**Method:**	Evaporate to dryness. Fume with Sulfuric Acid. Ignite and weigh as Li_2SO_4

***Instrument Analysis using ICP Spectrometer:** 999 µg/mL
via NIST SRM #3129a

We guarantee that our PerkinElmer TruQ Atomic Spectroscopy Standards are stable and accurate to ±0.5% of certified concentration until the expiration date, provided the standards are kept tightly capped and stored under normal laboratory conditions. This value is the sum of cumulative errors associated with the analytical determinations, pipetting, and diluting to final volume. For these solutions we use high purity acids, ASTM Type 1 water (18 megohm double deionized), and leached, triple-rinsed bottles. All glassware used is class A.

Certifying Officer: Y. Parikh
Yogesh Parikh, Senior Spectroscopist

PerkinElmer®

PerkinElmer Pure Plus

**PerkinElmer Number:**	N9303743	**Lot No:**	CL12-62MGY1
**Element and Matrix:**	1000 µg/mL Magnesium in 2% HNO3	**Certification Date:**	JAN - - 2021
**Starting Material:**	Magnesium Nitrate Hexahydrate	**Expiration Date:**	JUL 30 2022
**Starting Material Lot No:**	11151G		
**Density:**	1.013 g/mL @ 20°C		

Trace Metallic Impurities in the Actual Solution via ICP / ICP-MS Analysis:

Element	µg/L	Element	µg/L	Element	µg/L	Element	µg/L	Element	µg/L
Ag	<0.2	Cu	<0.2	La	0.02	Pt	<0.2	Tb	<0.02
Al	2	Dy	<0.04	Li	1	Rb	<0.08	Te	<0.7
As	<0.8	Er	<0.01	Lu	<0.01	Re	<0.01	Th	<0.01
Au	<0.7	Eu	<0.02	Mn	1	Rh	<0.02	Ti	2
B	2	Fe	1	Mo	<0.1	Ru	<0.5	Tl	<0.05
Ba	<0.1	Ga	<0.07	Na	30	Sb	<0.07	Tm	<0.03
Be	<0.2	Gd	<0.04	Nb	<0.04	Sc	0.3	U	<0.02
Bi	<0.03	Ge	<0.8	Nd	<0.01	Se	<6	V	0.2
Ca	40	Hf	<0.03	Ni	0.2	Si	<100	W	<0.07
Cd	<0.07	Hg	<0.1	P	<200	Sm	<0.03	Y	<0.03
Ce	0.04	Ho	<0.03	Pb	<0.3	Sn	<0.07	Yb	<0.01
Co	0.6	In	0.03	Pd	<0.2	Sr	<0.1	Zn	2
Cr	<1	Ir	<0.09	Pr	<0.01	Ta	<0.07	Zr	<0.1
Cs	0.08	K	4						

Traceability Documentation for Solution Standard:

Certified Value: 998 µg/mL ±5 µg/mL (refer to side 2)

Certified Value is Traceable to: NIST SRM #3131a

*** Classical Wet Assay:** 996 µg/mL

Method: EDTA titration using Eriochrome Black T as indicator. EDTA standardized against Pb(NO3)2 NIST SRM #928.

***Instrument Analysis using ICP Spectrometer:** 1000 µg/mL
via NIST SRM #3131a

We guarantee that our PerkinElmer Pure Plus Atomic Spectroscopy Standards are stable and accurate to ±0.5% of certified concentration until the expiration date, provided the standards are kept tightly capped and stored under normal laboratory conditions. This value is the sum of cumulative errors associated with the analytical determinations, pipetting, and diluting to final volume. For these solutions we use high purity acids, ASTM Type 1 water (18 megohm double deionized), and leached, triple-rinsed bottles. All glassware used is class A.

Certifying Officer: Y. Parikh
Yogesh Parikh, Senior Spectroscopist

PerkinElmer®

PerkinElmer Pure Plus

PerkinElmer Number: N9303744

Element and Matrix: 1000 µg/mL Manganese in 2% HNO_3

Starting Material: Manganese Metal

Starting Material Lot No: 03181D

Density: 1.011 g/mL @ 20°C

Lot No: CL11-204MNY1

Certification Date: MAR - - 2021

Expiration Date: SEP 3 0 2022

Trace Metallic Impurities in the Actual Solution via ICP / ICP-MS Analysis:

Element	µg/L	Element	µg/L	Element	µg/L	Element	µg/L	Element	µg/L
Ag	<0.3	Cu	1	La	0.03	Pt	<0.08	Tb	<0.01
Al	4	Dy	<0.01	Li	<0.3	Rb	<0.2	Te	<0.5
As	<0.2	Er	<0.01	Lu	<0.01	Re	<0.04	Th	<0.02
Au	<0.3	Eu	<0.02	Mg	60	Rh	<0.2	Ti	<0.3
B	8	Fe	3	Mo	<0.6	Ru	<0.3	Tl	<0.6
Ba	<0.04	Ga	<0.06	Na	6	Sb	<0.2	Tm	<0.01
Be	0.1	Gd	<0.01	Nb	<0.07	Sc	<0.2	U	0.01
Bi	<0.03	Ge	<3	Nd	<0.04	Se	<6	V	<0.03
Ca	20	Hf	<0.01	Ni	2	Si	<100	W	<0.2
Cd	<0.07	Hg	<0.4	P	<500	Sm	<0.04	Y	0.4
Ce	0.07	Ho	0.01	Pb	0.2	Sn	<0.04	Yb	<0.01
Co	3	In	<0.05	Pd	<0.08	Sr	2	Zn	2
Cr	<1	Ir	<0.05	Pr	0.02	Ta	<0.03	Zr	<0.2
Cs	<0.05	K	3						

Traceability Documentation for Solution Standard:

Certified Value: 1003 µg/mL ±5 µg/mL (refer to side 2)

Certified Value is Traceable to: NIST SRM #3132

*** Classical Wet Assay:** 1004 µg/mL

Method: EDTA titration using Eriochrome Black T as indicator. EDTA standardized against $Pb(NO_3)_2$ NIST SRM #928.

***Instrument Analysis using ICP Spectrometer:** 1002 µg/mL
via NIST SRM #3132

We guarantee that our PerkinElmer Pure Plus Atomic Spectroscopy Standards are stable and accurate to ±0.5% of certified concentration until the expiration date, provided the standards are kept tightly capped and stored under normal laboratory conditions. This value is the sum of cumulative errors associated with the analytical determinations, pipetting, and diluting to final volume. For these solutions we use high purity acids, ASTM Type 1 water (18 megohm double deionized), and leached, triple-rinsed bottles. All glassware used is class A.

Certifying Officer: Y. Parikh
Yogesh Parikh, Senior Spectroscopist

PerkinElmer Pure Plus
Atomic Spectroscopy Standard

ISO 9001 CERTIFIED

Certificate of Analysis

PerkinElmer Number: N9300253 **Lot No:** CL11-119HGY1

Element and Matrix: 10 µg/mL Mercury in 5% HNO3

Starting Material: Mercury Metal

Starting Material Lot No: 03141E

Certification Date: JUL -- 2020

Density: 1.024 g/mL @ 20°C

Expiration Date: JAN 30 2022

Trace Metallic Impurities in the Actual Solution via ICP / ICP-MS Analysis:

Element	µg/L	Element	µg/L	Element	µg/L	Element	µg/L	Element	µg/L
Ag	<0.2	Cu	2	Li	<0.3	Pt	<0.2	Tb	<0.01
Al	<0.9	Dy	<0.02	Lu	<0.01	Rb	<0.2	Te	<0.7
As	0.4	Er	<0.02	Mg	2	Re	<0.03	Th	<0.03
Au	<0.1	Eu	<0.02	Mn	<0.3	Rh	<0.03	Ti	<0.2
B	<0.4	Fe	<0.7	Mo	<0.2	Ru	<0.4	Tl	<0.8
Ba	<0.2	Ga	<0.07	Na	<1	Sb	<0.08	Tm	<0.01
Be	<0.5	Gd	<0.01	Nb	<0.2	Sc	<0.2	U	<0.01
Bi	<0.07	Ge	<0.7	Nd	<0.08	Se	<10	V	0.1
Ca	<0.2	Hf	<0.04	Ni	<0.6	Si	<50	W	<0.3
Cd	<0.08	Ho	<0.01	P	<100	Sm	<0.03	Y	<0.03
Ce	<0.02	In	<0.06	Pb	<0.2	Sn	<0.08	Yb	<0.02
Co	<0.1	Ir	<0.05	Pd	<0.1	Sr	0.1	Zn	0.8
Cr	0.2	K	4	Pr	<0.01	Ta	<0.04	Zr	<0.08
Cs	<0.04	La	<0.02						

Traceability Documentation for Solution Standard:

Certified Value: 10.0 µg/mL ±0.1 µg/mL (refer to side 2)

Certified Value is Traceable to: NIST SRM #3133

* **Classical Wet Assay:** 10.0 µg/mL

Method: This value was derived from dilution calculations of a Titrimetry analysis result of a Mercury concentrate. The concentrate was analyzed by Ammonium Thiocyanate titration using Ferric Nitrate as indicator.

* **Instrumental Analysis using OPTIMA 7300 DV ICP Spectrometer:** 10.0 µg/mL **via** NIST SRM #3133

We guarantee that our PerkinElmer Pure Plus Atomic Spectroscopy Standards are stable and accurate to ±1% of certified concentration until the expiration date, provided the standards are kept tightly capped and stored under normal laboratory conditions. This value is the sum of cumulative errors associated with the analytical determinations, pipetting, and diluting to final volume. For these solutions we use high purity acids, ASTM Type 1 water (18 megohm double deionized), and leached, triple-rinsed bottles. All glassware used is class A.

Certifying Officer: Y. Parikh

PerkinElmer®

PerkinElmer, Inc.
U.S.A. Tel: 1-203-925-4600
U.S.A. Toll Free: 1-800-762-4000

PerkinElmer®

PerkinElmer Pure

**PerkinElmer Number:**	N9303784		**Lot No:**	25-55MOY1
**Element and Matrix:**	1000 µg/mL Molybdenum in H2O		**Certification Date:**	JAN -- 2021
**Starting Material:**	Ammonium Molybdate(VI) Tetrahydrate		**Expiration Date:**	JUL 30 2022
**Starting Material Lot No:**	03201B			
**Density:**	1.000 g/mL @ 20°C			

Trace Metallic Impurities in the Actual Solution via ICP / ICP-MS Analysis:

Element	µg/mL	Element	µg/mL	Element	µg/mL	Element	µg/mL	Element	µg/mL
Ag	<0.003	Cu	0.003	La	<0.001	Pt	<0.001	Tb	<0.001
Al	0.003	Dy	<0.001	Li	<0.001	Rb	<0.001	Te	0.004
As	0.03	Er	<0.001	Lu	<0.001	Re	0.004	Th	<0.001
Au	<0.001	Eu	<0.001	Mg	<0.001	Rh	<0.001	Ti	0.005
B	<0.002	Fe	<0.005	Mn	0.006	Ru	0.008	Tl	<0.001
Ba	0.007	Ga	<0.001	Na	<0.01	Sb	<0.001	Tm	<0.001
Be	<0.001	Gd	<0.001	Nb	<0.001	Sc	<0.001	U	<0.001
Bi	<0.001	Ge	0.001	Nd	<0.001	Se	<0.001	V	<0.001
Ca	0.03	Hf	<0.001	Ni	<0.001	Si	<0.05	W	0.07
Cd	<0.02	Hg	<0.001	P	<0.1	Sm	<0.001	Y	<0.001
Ce	0.002	Ho	<0.001	Pb	<0.001	Sn	<0.001	Yb	<0.001
Co	<0.001	In	<0.001	Pd	0.02	Sr	0.002	Zn	<0.01
Cr	0.004	Ir	<0.001	Pr	0.002	Ta	<0.001	Zr	<0.001
Cs	<0.001	K	0.03						

Traceability Documentation for Solution Standard:

Certified Value: 1001 µg/mL ±5 µg/mL (refer to side 2)

Certified Value is Traceable to: NIST SRM #3134

*** Classical Wet Assay:** 1001 µg/mL

Method: Precipitation using 8-Hydroxy Quinoline. Filter, dry, and weigh as MoO2(C9H6NO)2.

***Instrument Analysis using ICP Spectrometer:** 1000 µg/mL
via NIST SRM #3134

We guarantee that our PerkinElmer Pure Atomic Spectroscopy Standards are stable and accurate to ±0.5% of certified concentration until the expiration date, provided the standards are kept tightly capped and stored under normal laboratory conditions. This value is the sum of cumulative errors associated with the analytical determinations, pipetting, and diluting to final volume. For these solutions we use high purity acids, ASTM Type 1 water (18 megohm double deionized), and leached, triple-rinsed bottles. All glassware used is class A.

Certifying Officer: Y. Parikh
Yogesh Parikh, Senior Spectroscopist

PerkinElmer®

PerkinElmer Pure Plus

**PerkinElmer Number:**	N9303747	**Lot No:**	CL12-43NIY1
**Element and Matrix:**	1000 µg/mL Nickel in 2% HNO3	**Certification Date:**	NOV - - 2020
**Starting Material:**	Nickel Metal	**Expiration Date:**	MAY 30 2022
**Starting Material Lot No:**	07181F		
**Density:**	1.011 g/mL @ 20°C		

Trace Metallic Impurities in the Actual Solution via ICP / ICP-MS Analysis:

Element	µg/L	Element	µg/L	Element	µg/L	Element	µg/L	Element	µg/L
Ag	<0.06	Cu	<0.7	La	<0.04	Pt	<0.2	Tb	<0.01
Al	0.9	Dy	<0.01	Li	<0.3	Rb	<0.3	Te	<0.5
As	2	Er	<0.01	Lu	<0.01	Re	0.2	Th	<0.02
Au	<0.09	Eu	<0.01	Mg	<0.4	Rh	<0.5	Ti	<0.4
B	<2	Fe	3	Mn	<0.3	Ru	<0.2	Tl	<0.7
Ba	0.02	Ga	<0.02	Mo	<0.6	Sb	1	Tm	<0.01
Be	<0.4	Gd	<0.01	Na	0.7	Sc	<0.6	U	<0.01
Bi	<0.03	Ge	<0.7	Nb	<0.02	Se	<30	V	<0.3
Ca	3	Hf	<0.01	Nd	<0.05	Si	<100	W	<0.1
Cd	<0.2	Hg	<0.8	P	<500	Sm	<0.02	Y	<0.02
Ce	0.06	Ho	<0.01	Pb	<0.01	Sn	<0.2	Yb	<0.01
Co	5	In	<0.02	Pd	<0.1	Sr	<0.1	Zn	1
Cr	0.9	Ir	0.6	Pr	<0.02	Ta	<0.05	Zr	<0.08
Cs	<0.04	K	2						

Traceability Documentation for Solution Standard:

**Certified Value:**	998 µg/mL ±5 µg/mL (refer to side 2)
**Certified Value is Traceable to:**	NIST SRM #3136
*** Classical Wet Assay:**	997 µg/mL
**Method:**	EDTA titration using Murexide as indicator. EDTA standardized against Pb(NO3)2 NIST SRM #92<br>8

***Instrument Analysis using ICP Spectrometer:** 998 µg/mL
via NIST SRM #3136

We guarantee that our PerkinElmer Pure Plus Atomic Spectroscopy Standards are stable and accurate to ±0.5% of certified concentration until the expiration date, provided the standards are kept tightly capped and stored under normal laboratory conditions. This value is the sum of cumulative errors associated with the analytical determinations, pipetting, and diluting to final volume. For these solutions we use high purity acids, ASTM Type 1 water (18 megohm double deionized), and leached, triple-rinsed bottles. All glassware used is class A.

Certifying Officer: Y. Parikh
Yogesh Parikh, Senior Spectroscopist

PerkinElmer®

PerkinElmer Pure

**PerkinElmer Number:**	N9303789	**Lot No:**	25-17PDY1
**Element and Matrix:**	1000 µg/mL Palladium in 10% HCl	**Certification Date:**	FEB - - 2021
**Starting Material:**	Palladium Metal	**Expiration Date:**	AUG 30 2022
**Starting Material Lot No:**	04191D		
**Density:**	1.021 g/mL @ 20°C		

Trace Metallic Impurities in the Actual Solution via ICP / ICP-MS Analysis:

Element	µg/mL	Element	µg/mL	Element	µg/mL	Element	µg/mL	Element	µg/mL
Ag	0.001	Cu	0.001	La	<0.001	Pt	<0.001	Tb	<0.001
Al	0.004	Dy	<0.001	Li	<0.001	Rb	<0.001	Te	<0.001
As	<0.003	Er	<0.001	Lu	<0.001	Re	<0.001	Th	<0.001
Au	0.02	Eu	<0.001	Mg	0.001	Rh	0.002	Ti	<0.001
B	<0.001	Fe	0.2	Mn	0.001	Ru	<0.001	Tl	0.002
Ba	<0.001	Ga	<0.001	Mo	0.005	Sb	<0.001	Tm	<0.001
Be	<0.001	Gd	<0.001	Na	0.009	Sc	<0.001	U	<0.001
Bi	<0.001	Ge	<0.001	Nb	<0.001	Se	<0.006	V	<0.001
Ca	0.06	Hf	<0.001	Nd	<0.001	Si	<0.2	W	<0.001
Cd	<0.001	Hg	0.005	Ni	0.03	Sm	<0.001	Y	<0.001
Ce	<0.001	Ho	<0.001	P	<0.5	Sn	<0.001	Yb	<0.001
Co	<0.001	In	<0.001	Pb	0.001	Sr	<0.001	Zn	0.03
Cr	0.02	Ir	<0.001	Pr	<0.001	Ta	<0.001	Zr	<0.001
Cs	<0.001	K	0.008						

Traceability Documentation for Solution Standard:

**Certified Value:**	998 µg/mL ±5 µg/mL (refer to side 2)
**Certified Value is Traceable to:**	NIST SRM #3138
*** Classical Wet Assay:**	998 µg/mL
**Method:**	Precipitation using Glyoxime. Filter, dry, and weigh as Pd(C4H7O2N2)2

***Instrument Analysis using ICP Spectrometer:** 998 µg/mL
via NIST SRM #3138

We guarantee that our PerkinElmer Pure Atomic Spectroscopy Standards are stable and accurate to ±0.5% of certified concentration until the expiration date, provided the standards are kept tightly capped and stored under normal laboratory conditions. This value is the sum of cumulative errors associated with the analytical determinations, pipetting, and diluting to final volume. For these solutions we use high purity acids, ASTM Type 1 water (18 megohm double deionized), and leached, triple-rinsed bottles. All glassware used is class A.

Certifying Officer: Y. Parikh
Yogesh Parikh, Senior Spectroscopist

PerkinElmer

PerkinElmer Pure

**PerkinElmer Number:**	N9303791	**Lot No:**	25-85PTY1
**Element and Matrix:**	1000 µg/mL Platinum in 10% HCl	**Certification Date:**	APR - - 2021
**Starting Material:**	Platinum Metal	**Expiration Date:**	OCT 30 2022
**Starting Material Lot No:**	06201F		
**Density:**	1.021 g/mL @ 20°C		

Trace Metallic Impurities in the Actual Solution via ICP / ICP-MS Analysis:

Element	µg/mL	Element	µg/mL	Element	µg/mL	Element	µg/mL	Element	µg/mL
Ag	0.001	Cu	<0.001	La	<0.001	Pr	<0.001	Tb	<0.001
Al	0.006	Dy	<0.001	Li	0.2	Rb	<0.001	Te	<0.001
As	<0.001	Er	<0.001	Lu	<0.001	Re	<0.001	Th	<0.001
Au	0.03	Eu	<0.001	Mg	<0.001	Rh	0.008	Ti	<0.005
B	<0.005	Fe	0.1	Mn	<0.001	Ru	<0.001	Tl	0.006
Ba	<0.001	Ga	<0.001	Mo	0.003	Sb	<0.001	Tm	<0.001
Be	<0.001	Gd	<0.001	Na	0.1	Sc	<0.001	U	<0.001
Bi	0.002	Ge	<0.001	Nb	<0.001	Se	0.008	V	0.002
Ca	0.04	Hf	<0.001	Nd	<0.001	Si	<0.1	W	<0.001
Cd	<0.001	Hg	0.004	Ni	0.02	Sm	<0.001	Y	<0.001
Ce	<0.001	Ho	<0.001	P	<0.1	Sn	0.004	Yb	<0.001
Co	<0.001	In	<0.001	Pb	0.009	Sr	<0.001	Zn	0.007
Cr	0.03	Ir	0.001	Pd	0.009	Ta	<0.001	Zr	<0.001
Cs	<0.001	K	<0.5						

Traceability Documentation for Solution Standard:

**Certified Value:**	1002 µg/mL ±5 µg/mL (refer to side 2)
**Certified Value is Traceable to:**	NIST SRM #3140
*** Classical Wet Assay:**	1005 µg/mL
**Method:**	Precipitation using Ammonium Chloride. Filter, ignite, and weigh as Platinum metal.

***Instrument Analysis using ICP Spectrometer:** 999 µg/mL
via NIST SRM #3140

We guarantee that our PerkinElmer Pure Atomic Spectroscopy Standards are stable and accurate to ±0.5% of certified concentration until the expiration date, provided the standards are kept tightly capped and stored under normal laboratory conditions. This value is the sum of cumulative errors associated with the analytical determinations, pipetting, and diluting to final volume. For these solutions we use high purity acids, ASTM Type 1 water (18 megohm double deionized), and leached, triple-rinsed bottles. All glassware used is class A.

Certifying Officer: Y. Parikh
Yogesh Parikh, Senior Spectroscopist

PerkinElmer®

PerkinElmer Pure

PerkinElmer Number: N9300171

Element and Matrix: 1000 µg/mL Silver in 2% HNO_3

Starting Material: Silver nitrate

Starting Material Lot No: 11191B

Density: 1.010 g/mL @ 20°C

Lot No: 25-57AGY1

Certification Date: AUG - - 2021

Expiration Date: FEB 2 8 2023

Trace Metallic Impurities in the Actual Solution via ICP / ICP-MS Analysis:

Element	µg/mL	Element	µg/mL	Element	µg/mL	Element	µg/mL	Element	µg/mL
Al	0.005	Dy	<0.001	Li	<0.001	Pt	<0.001	Tb	<0.001
As	<0.001	Er	<0.001	Lu	<0.001	Rb	<0.001	Te	<0.002
Au	<0.001	Eu	<0.001	Mg	0.001	Re	<0.001	Th	<0.001
B	0.001	Fe	0.004	Mn	<0.001	Rh	0.01	Ti	<0.001
Ba	0.001	Ga	<0.001	Mo	0.004	Ru	<0.001	Tl	<0.005
Be	<0.001	Gd	<0.001	Na	0.02	Sb	<0.001	Tm	<0.001
Bi	<0.001	Ge	<0.001	Nb	<0.001	Sc	<0.001	U	<0.001
Ca	0.02	Hf	<0.001	Nd	<0.001	Se	<0.006	V	<0.001
Cd	<0.001	Hg	<0.001	Ni	<0.001	Si	<0.1	W	<0.001
Ce	<0.001	Ho	<0.001	P	<0.1	Sm	<0.001	Y	<0.001
Co	<0.001	In	<0.001	Pb	<0.001	Sn	0.002	Yb	<0.001
Cr	<0.001	Ir	<0.001	Pd	0.02	Sr	<0.001	Zn	0.09
Cs	<0.001	K	0.006	Pr	<0.001	Ta	<0.001	Zr	0.1
Cu	<0.001	La	<0.001						

Traceability Documentation for Solution Standard:

Certified Value: 1001 µg/mL ±5 µg/mL (refer to side 2)

Certified Value is Traceable to: NIST SRM #3151

*** Classical Wet Assay:** 1000 µg/mL

Method: Precipitation using Hydrochloric Acid. Filter, dry, and weigh as AgCl.

***Instrument Analysis using ICP Spectrometer:** 1002 µg/mL
via NIST SRM #3151

We guarantee that our PerkinElmer Pure Atomic Spectroscopy Standards are stable and accurate to ±0.5% of certified concentration until the expiration date, provided the standards are kept tightly capped and stored under normal laboratory conditions. This value is the sum of cumulative errors associated with the analytical determinations, pipetting, and diluting to final volume. For these solutions we use high purity acids, ASTM Type 1 water (18 megohm double deionized), and leached, triple-rinsed bottles. All glassware used is class A.

Certifying Officer: Y. Parikh
Yogesh Parikh, Senior Spectroscopist

PerkinElmer

PerkinElmer Pure

PerkinElmer Number: N9300170
Element and Matrix: 1000 µg/mL Thallium in 2% HNO3
Starting Material: Thallium(I) Nitrate
Starting Material Lot No: 03191C
Density: 1.010 g/mL @ 20°C

Lot No: 25-29TLY1
Certification Date: MAY - - 2021
Expiration Date: NOV 30 2022

Trace Metallic Impurities in the Actual Solution via ICP / ICP-MS Analysis:

Element	µg/mL	Element	µg/mL	Element	µg/mL	Element	µg/mL	Element	µg/mL
Ag	0.002	Cu	<0.001	La	<0.001	Pr	<0.001	Ta	<0.001
Al	<0.002	Dy	<0.001	Li	<0.002	Pt	<0.001	Tb	<0.001
As	<0.001	Er	<0.001	Lu	<0.001	Rb	<0.001	Te	<0.001
Au	<0.001	Eu	<0.001	Mg	<0.001	Re	<0.001	Th	<0.001
B	<0.001	Fe	0.003	Mn	<0.001	Rh	<0.001	Ti	<0.001
Ba	<0.001	Ga	<0.001	Mo	<0.001	Ru	<0.001	Tm	<0.001
Be	<0.001	Gd	<0.001	Na	0.007	Sb	<0.001	U	<0.001
Bi	<0.001	Ge	<0.001	Nb	<0.001	Sc	<0.001	V	<0.001
Ca	0.007	Hf	<0.001	Nd	<0.001	Se	<0.001	W	<0.001
Cd	<0.001	Hg	<0.001	Ni	<0.001	Si	<0.05	Y	<0.001
Ce	<0.001	Ho	<0.001	P	<0.1	Sm	<0.001	Yb	<0.001
Co	<0.001	In	<0.001	Pb	<0.001	Sn	<0.001	Zn	0.005
Cr	0.001	Ir	<0.001	Pd	<0.001	Sr	<0.001	Zr	<0.001
Cs	<0.001	K	0.04						

Traceability Documentation for Solution Standard:

Certified Value: 1000 µg/mL ±5 µg/mL (refer to side 2)

Certified Value is Traceable to: NIST SRM #3158

*** Classical Wet Assay:** 1001 µg/mL

Method: Precipitation using Potassium Chromate. Filter, dry, and weigh as TI2CrO4

***Instrument Analysis using ICP Spectrometer:** 999 µg/mL
via NIST SRM #3158

We guarantee that our PerkinElmer Pure Atomic Spectroscopy Standards are stable and accurate to ±0.5% of certified concentration until the expiration date, provided the standards are kept tightly capped and stored under normal laboratory conditions. This value is the sum of cumulative errors associated with the analytical determinations, pipetting, and diluting to final volume. For these solutions we use high purity acids, ASTM Type 1 water (18 megohm double deionized), and leached, triple-rinsed bottles. All glassware used is class A.

Certifying Officer: Y. Parikh
Yogesh Parikh, Senior Spectroscopist

PerkinElmer

PerkinElmer Pure

PerkinElmer Number: N9303808
Element and Matrix: 1000 µg/mL Vanadium in 2% HNO3
Starting Material: Ammonium Metavanadate(V)
Starting Material Lot No: 01181C
Density: 1.012 g/mL @ 20°C

Lot No: 25-30VY1
Certification Date: SEP - - 2020
Expiration Date: MAR 3 0 2022

Trace Metallic Impurities in the Actual Solution via ICP / ICP-MS Analysis:

Element	µg/mL	Element	µg/mL	Element	µg/mL	Element	µg/mL	Element	µg/mL
Ag	<0.001	Cu	<0.001	La	<0.001	Pr	<0.001	Ta	<0.001
Al	0.003	Dy	<0.001	Li	<0.001	Pt	<0.001	Tb	<0.001
As	<0.001	Er	<0.001	Lu	<0.001	Rb	<0.001	Te	<0.001
Au	<0.001	Eu	<0.001	Mg	0.004	Re	<0.001	Th	<0.001
B	<0.003	Fe	0.06	Mn	0.003	Rh	<0.001	Ti	<0.002
Ba	0.001	Ga	<0.001	Mo	0.003	Ru	<0.001	Tl	0.02
Be	<0.001	Gd	<0.001	Na	0.02	Sb	<0.001	Tm	<0.001
Bi	<0.001	Ge	<0.001	Nb	<0.001	Sc	<0.001	U	<0.001
Ca	0.03	Hf	<0.001	Nd	<0.001	Se	<0.08	W	<0.001
Cd	<0.001	Hg	<0.001	Ni	0.002	Si	<0.1	Y	<0.001
Ce	<0.001	Ho	<0.001	P	<0.2	Sm	<0.001	Yb	<0.001
Co	<0.001	In	<0.001	Pb	0.003	Sn	<0.001	Zn	0.003
Cr	0.006	Ir	<0.001	Pd	<0.001	Sr	<0.001	Zr	0.001
Cs	0.004	K	0.02						

Traceability Documentation for Solution Standard:

Certified Value: 999 µg/mL ±5 µg/mL (refer to side 2)
Certified Value is Traceable to: NIST SRM #3165
*** Classical Wet Assay:** 997 µg/mL
Method: Evaporate to dryness. Fume with Nitric acid. Ignite and weigh as V2O5.

***Instrument Analysis using ICP Spectrometer:** 1001 µg/mL
via NIST SRM #3165

We guarantee that our PerkinElmer Pure Atomic Spectroscopy Standards are stable and accurate to ±0.5% of certified concentration until the expiration date, provided the standards are kept tightly capped and stored under normal laboratory conditions. This value is the sum of cumulative errors associated with the analytical determinations, pipetting, and diluting to final volume. For these solutions we use high purity acids, ASTM Type 1 water (18 megohm double deionized), and leached, triple-rinsed bottles. All glassware used is class A.

Certifying Officer: Y. Parikh
Yogesh Parikh, Senior Spectroscopist

PerkinElmer®

PerkinElmer Pure Plus

PerkinElmer Number: N9303758

Element and Matrix: 1000 µg/mL Zinc in 2% HNO3

Starting Material: Zinc Metal

Starting Material Lot No: 09161G

Density: 1.011 g/mL @ 20°C

Lot No: CL11-196ZNY1

Certification Date: OCT - - 2020

Expiration Date: APR 30 2022

Trace Metallic Impurities in the Actual Solution via ICP / ICP-MS Analysis:

Element	µg/L	Element	µg/L	Element	µg/L	Element	µg/L	Element	µg/L
Ag	<0.4	Cu	<0.2	La	<0.03	Pr	<0.01	Ta	<0.05
Al	2	Dy	<0.01	Li	0.2	Pt	<0.2	Tb	<0.01
As	<0.1	Er	<0.01	Lu	<0.01	Rb	<0.09	Te	0.6
Au	<0.05	Eu	<0.01	Mg	<0.3	Re	<0.05	Th	<0.02
B	<0.3	Fe	<0.2	Mn	0.7	Rh	<0.6	Ti	0.3
Ba	<0.06	Ga	0.2	Mo	<0.3	Ru	<0.3	Tl	<0.5
Be	<0.2	Gd	<0.03	Na	4	Sb	<0.3	Tm	<0.01
Bi	0.06	Ge	0.01	Nb	<0.07	Sc	<0.3	U	<0.01
Ca	<4	Hf	<0.01	Nd	<0.01	Se	<10	V	<0.1
Cd	3	Hg	<0.2	Ni	0.2	Si	<100	W	<0.2
Ce	<0.02	Ho	<0.01	P	<200	Sm	<0.01	Y	<0.02
Co	<0.2	In	<0.08	Pb	1	Sn	0.2	Yb	<0.01
Cr	<0.5	Ir	<0.2	Pd	<0.2	Sr	<0.04	Zr	0.04
Cs	<0.08	K	0.5						

Traceability Documentation for Solution Standard:

Certified Value: 1000 µg/mL ±5 µg/mL (refer to side 2)

Certified Value is Traceable to: NIST SRM #3168a

*** Classical Wet Assay:** 999 µg/mL

Method: EDTA titration using Eriochrome Black-T as indicator. EDTA standardized against Pb(NO3)2 NIST SRM #928.

***Instrument Analysis using ICP Spectrometer:** 1000 µg/mL
via NIST SRM #3168a

We guarantee that our PerkinElmer Pure Plus Atomic Spectroscopy Standards are stable and accurate to ±0.5% of certified concentration until the expiration date, provided the standards are kept tightly capped and stored under normal laboratory conditions. This value is the sum of cumulative errors associated with the analytical determinations, pipetting, and diluting to final volume. For these solutions we use high purity acids, ASTM Type 1 water (18 megohm double deionized), and leached, triple-rinsed bottles. All glassware used is class A.

Certifying Officer: Y. Parikh
Yogesh Parikh, Senior Spectroscopist

PerkinElmer

PerkinElmer Pure

**PerkinElmer Number:**	N9300207	**Lot No:**	25-80SBY1
**Element and Matrix:**	1000 µg/mL Antimony in H2O/0.6% Tart.Acid/Tr.HNO3	**Certification Date:**	APR - - 2021
**Starting Material:**	Antimony Metal	**Expiration Date:**	OCT 30 2022
**Starting Material Lot No:**	05171E		
**Density:**	1.001 g/mL @ 20°C		

Trace Metallic Impurities in the Actual Solution via ICP / ICP-MS Analysis:

Element	µg/mL	Element	µg/mL	Element	µg/mL	Element	µg/mL	Element	µg/mL
Ag	<0.001	Cu	<0.001	La	0.02	Pr	<0.001	Tb	<0.001
Al	0.006	Dy	0.002	Li	<0.001	Pt	<0.001	Te	<0.001
As	<0.001	Er	<0.001	Lu	<0.001	Rb	<0.001	Th	<0.001
Au	<0.001	Eu	<0.001	Mg	0.001	Re	<0.001	Ti	<0.001
B	<0.003	Fe	0.006	Mn	<0.001	Rh	<0.001	Tl	<0.001
Ba	0.002	Ga	<0.001	Mo	<0.001	Ru	<0.001	Tm	<0.001
Be	<0.001	Gd	<0.001	Na	0.006	Sc	<0.001	U	<0.001
Bi	<0.001	Ge	<0.001	Nb	<0.001	Se	<0.01	V	<0.001
Ca	0.01	Hf	<0.001	Nd	<0.001	Si	<0.2	W	<0.001
Cd	<0.001	Hg	<0.001	Ni	<0.001	Sm	<0.001	Y	<0.001
Ce	<0.001	Ho	<0.001	P	<0.2	Sn	<0.001	Yb	<0.001
Co	<0.001	In	<0.001	Pb	<0.001	Sr	<0.001	Zn	0.005
Cr	<0.001	Ir	<0.001	Pd	<0.001	Ta	<0.001	Zr	<0.001
Cs	<0.001	K	0.02						

Traceability Documentation for Solution Standard:

**Certified Value:**	999 µg/mL ±5 µg/mL (refer to side 2)
**Certified Value is Traceable to:**	NIST SRM #3102a
*** Classical Wet Assay:**	997 µg/mL
**Method:**	Evaporate to dryness. Fume with Nitric Acid. Ignite and weigh as Sb2O4.

***Instrument Analysis using ICP Spectrometer:** 1000 µg/mL
via NIST SRM #3102a

We guarantee that our PerkinElmer Pure Atomic Spectroscopy Standards are stable and accurate to ±0.5% of certified concentration until the expiration date, provided the standards are kept tightly capped and stored under normal laboratory conditions. This value is the sum of cumulative errors associated with the analytical determinations, pipetting, and diluting to final volume. For these solutions we use high purity acids, ASTM Type 1 water (18 megohm double deionized), and leached, triple-rinsed bottles. All glassware used is class A.

Certifying Officer: Y. Parikh
Yogesh Parikh, Senior Spectroscopist
