# Supplementary material for: Elemental Analysis and In Vitro Evaluation of Antibacterial and Antifungal Activities of Usnea barbata (L.) Weber ex F.H. Wigg from Călimani Mountains, Romania
Source: Plants (Basel). 2021 Dec 23;11(1):32. doi: 10.3390/plants11010032 (PMC8747648; doi:10.3390/plants11010032)
Supplement: Supplementary file 1 [file plants-11-00032-s001.zip › Figure S1. Calibrations curves for 23 elements analyzed in U. barbata dried lichen using ICP-MS.pdf]

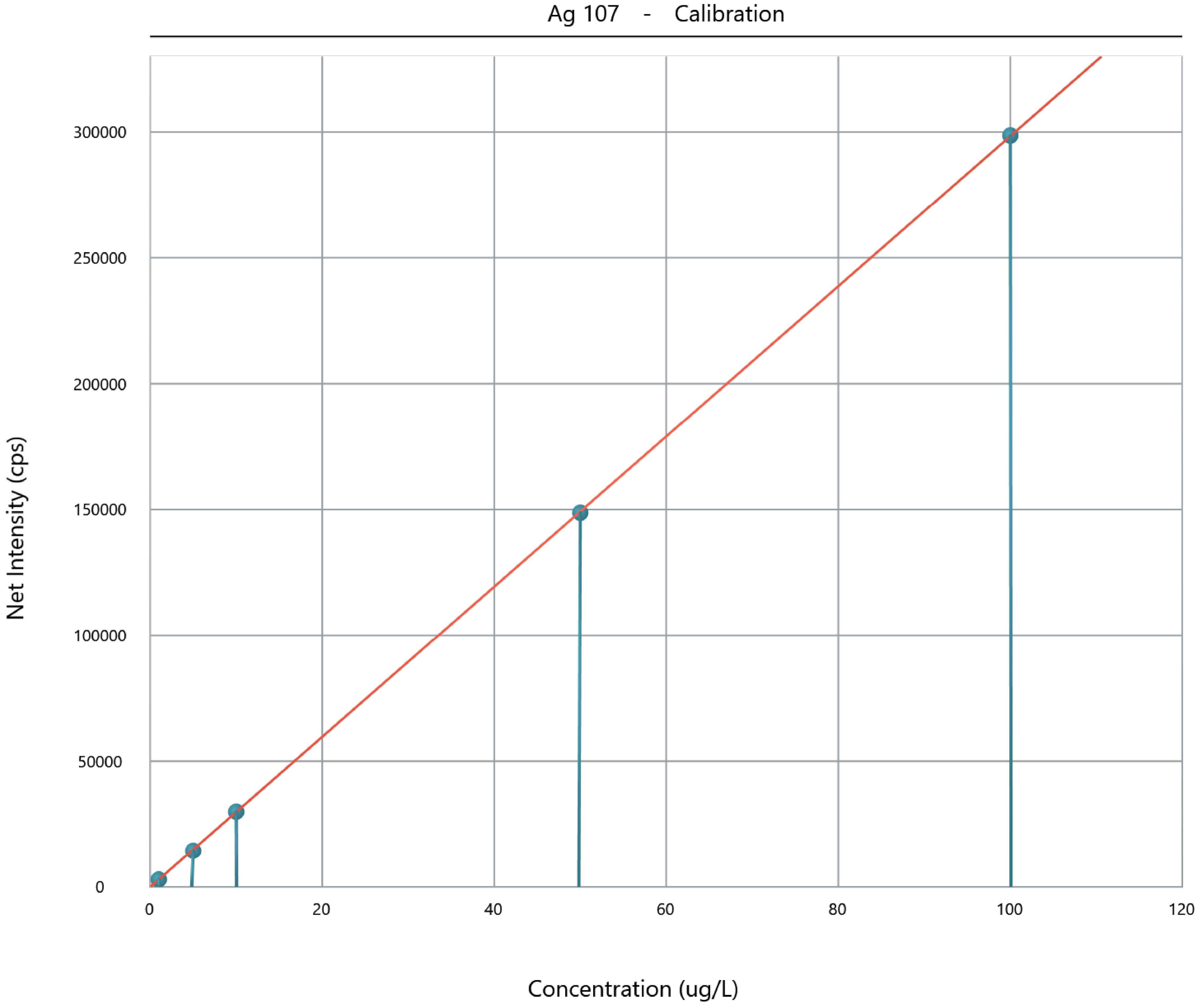

Statistics

Eqn. :	y = 2984x + 0	
Cor.Coeff. :	0.999997	
BEC :	0.069488	ug/L
DL :	0.035921	ug/L

Calibration Table

	Net Intensity Ag 107 (cps)	Apparent Conc. Ag 107 (ug/L)
Blank	207.3	
Cal. Std.1	2979.0	0.998
Cal. Std.2	14422.8	4.834
Cal. Std.3	29962.2	10.042
Cal. Std.4	148698.0	49.836
Cal. Std.5	298633.6	100.086

Al 27 - Calibration

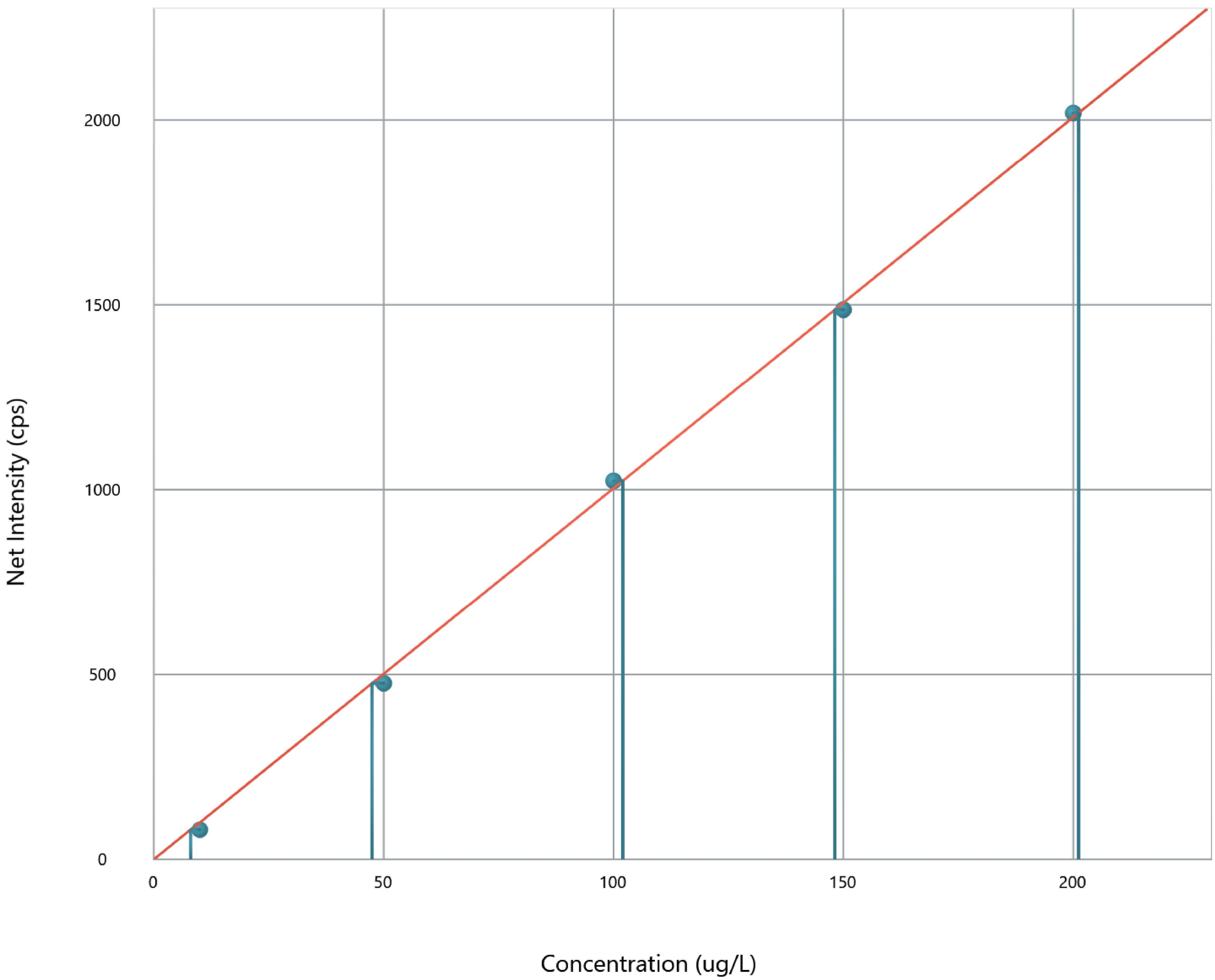

Statistics

Eqn. :	y = 10x + 0	
Cor.Coeff. :	0.999872	
BEC :	5.845140	ug/L
DL :	1.646212	ug/L

Calibration Table

	Net Intensity Al 27 (cps)	Apparent Conc. Al 27 (ug/L)
Blank	58.7	
Cal. Std.1	80.3	8.004
Cal. Std.2	476.3	47.459
Cal. Std.3	1023.7	101.995
Cal. Std.4	1486.8	148.129
Cal. Std.5	2018.8	201.141

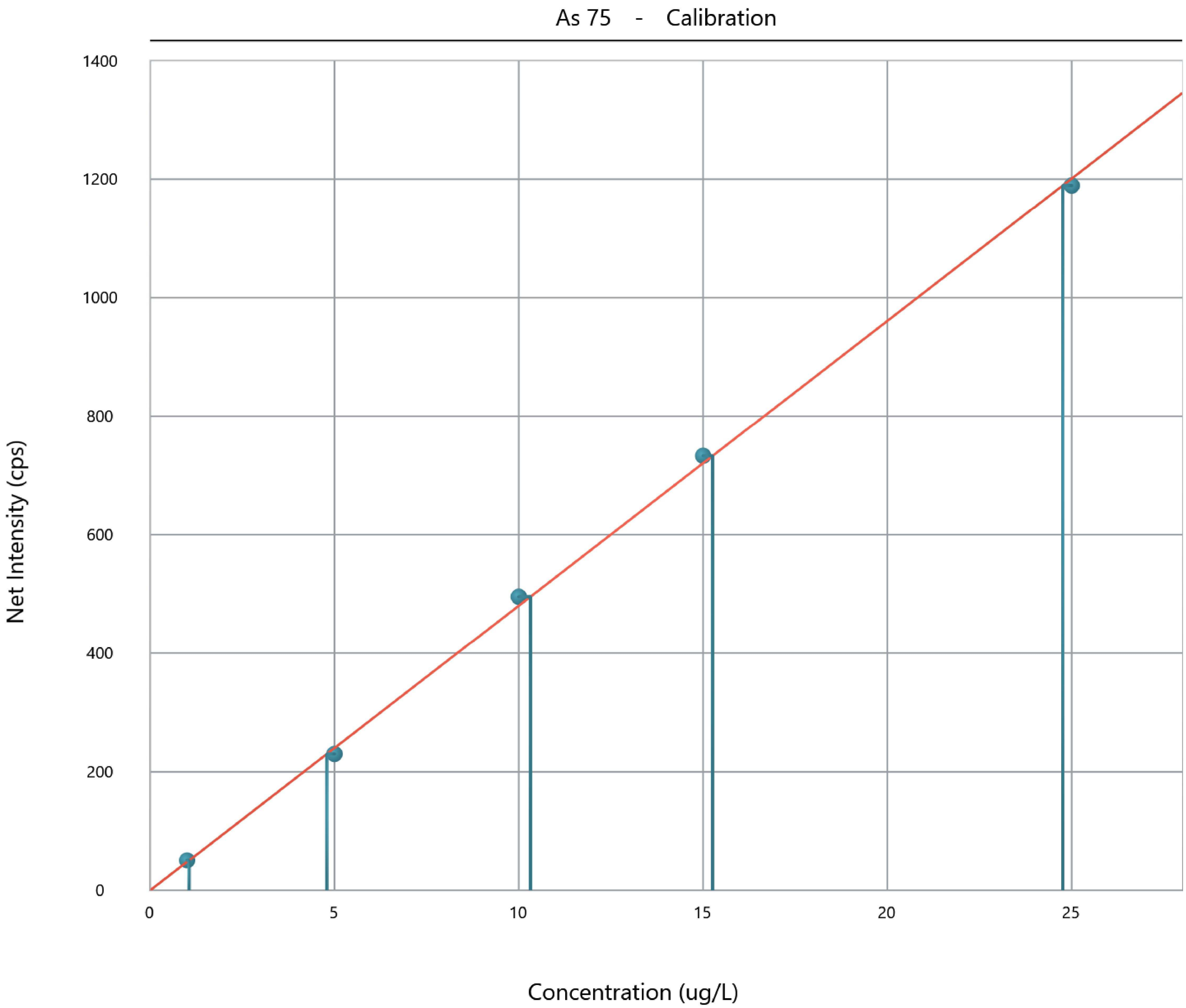

Statistics

Eqn. :	y = 48x + 0	
Cor.Coeff. :	0.999861	
BEC :	0.152644	ug/L
DL :	0.036053	ug/L

Calibration Table

	Net Intensity As 75 (cps)	Apparent Conc. As 75 (ug/L)
Blank	7.3	
Cal. Std.1	50.7	1.055
Cal. Std.2	230.3	4.794
Cal. Std.3	495.7	10.318
Cal. Std.4	733.0	15.258
Cal. Std.5	1189.4	24.757

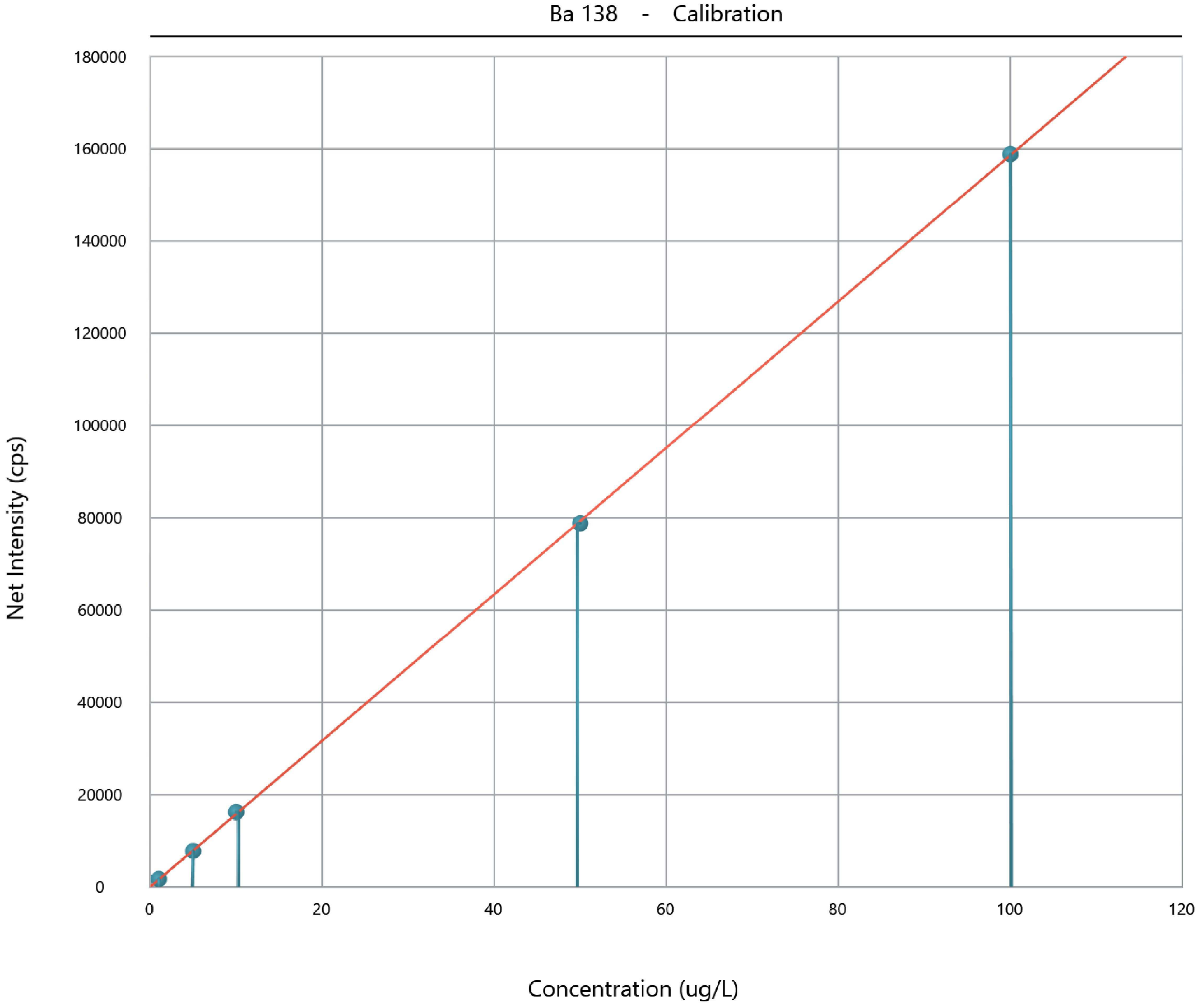

Statistics

Eqn. :	y = 1586x + 0	
Cor.Coeff. :	0.999992	
BEC :	0.070481	ug/L
DL :	0.022877	ug/L

Calibration Table

	Net Intensity Ba 138 (cps)	Apparent Conc. Ba 138 (ug/L)
Blank	111.8	
Cal. Std.1	1701.1	1.072
Cal. Std.2	7814.3	4.926
Cal. Std.3	16291.6	10.270
Cal. Std.4	78805.6	49.680
Cal. Std.5	158843.2	100.136

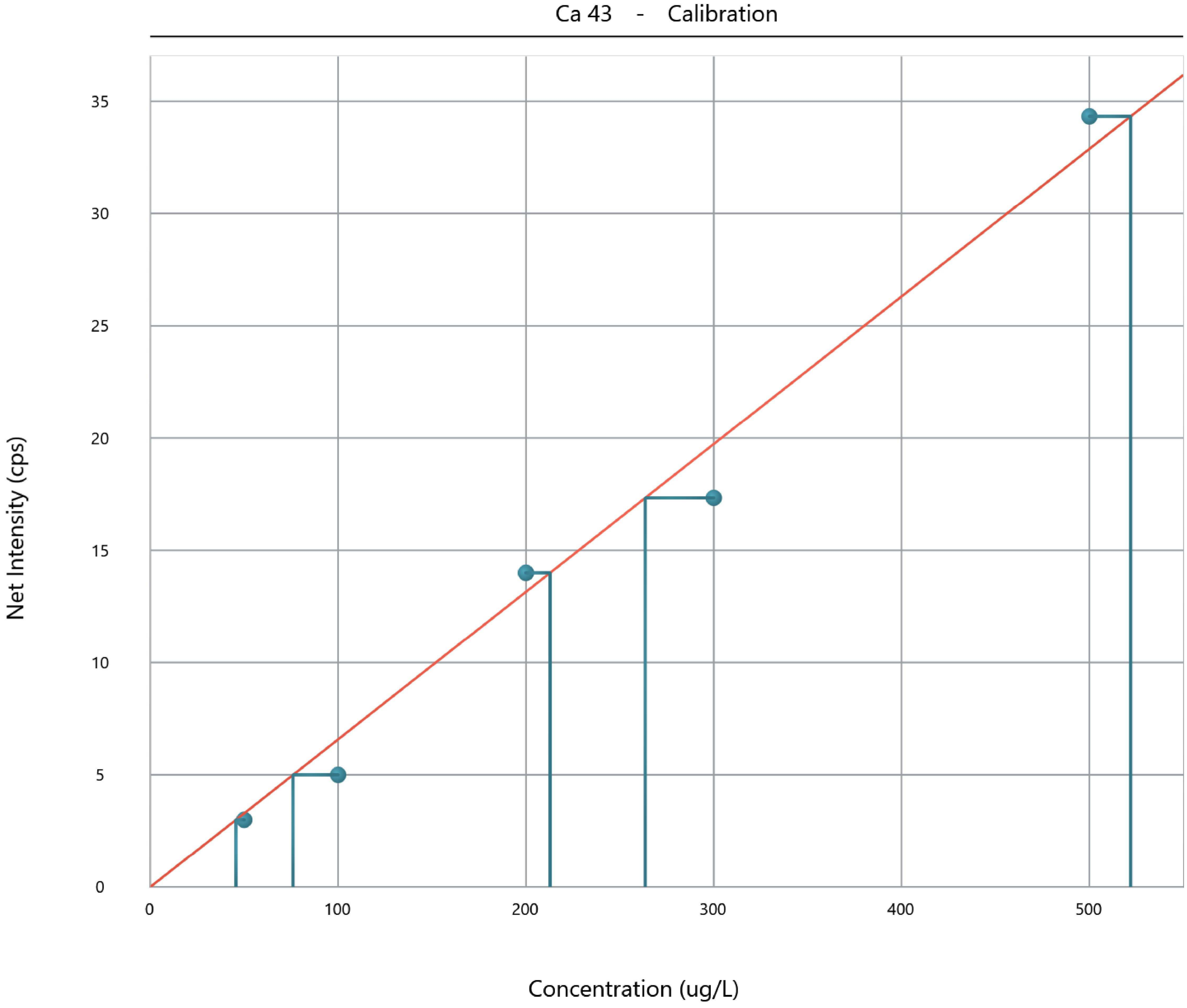

Statistics

Eqn. :	y = 0x + 0	
Cor.Coeff. :	0.996738	
BEC :	50.677799	ug/L
DL :	94.944832	ug/L

Calibration Table

	Net Intensity Ca 43 (cps)	Apparent Conc. Ca 43 (ug/L)
Blank	3.3	
Cal. Std.1	3.0	45.610
Cal. Std.2	5.0	76.017
Cal. Std.3	14.0	212.847
Cal. Std.4	17.3	263.525
Cal. Std.5	34.3	521.982

Cd 111 - Calibration

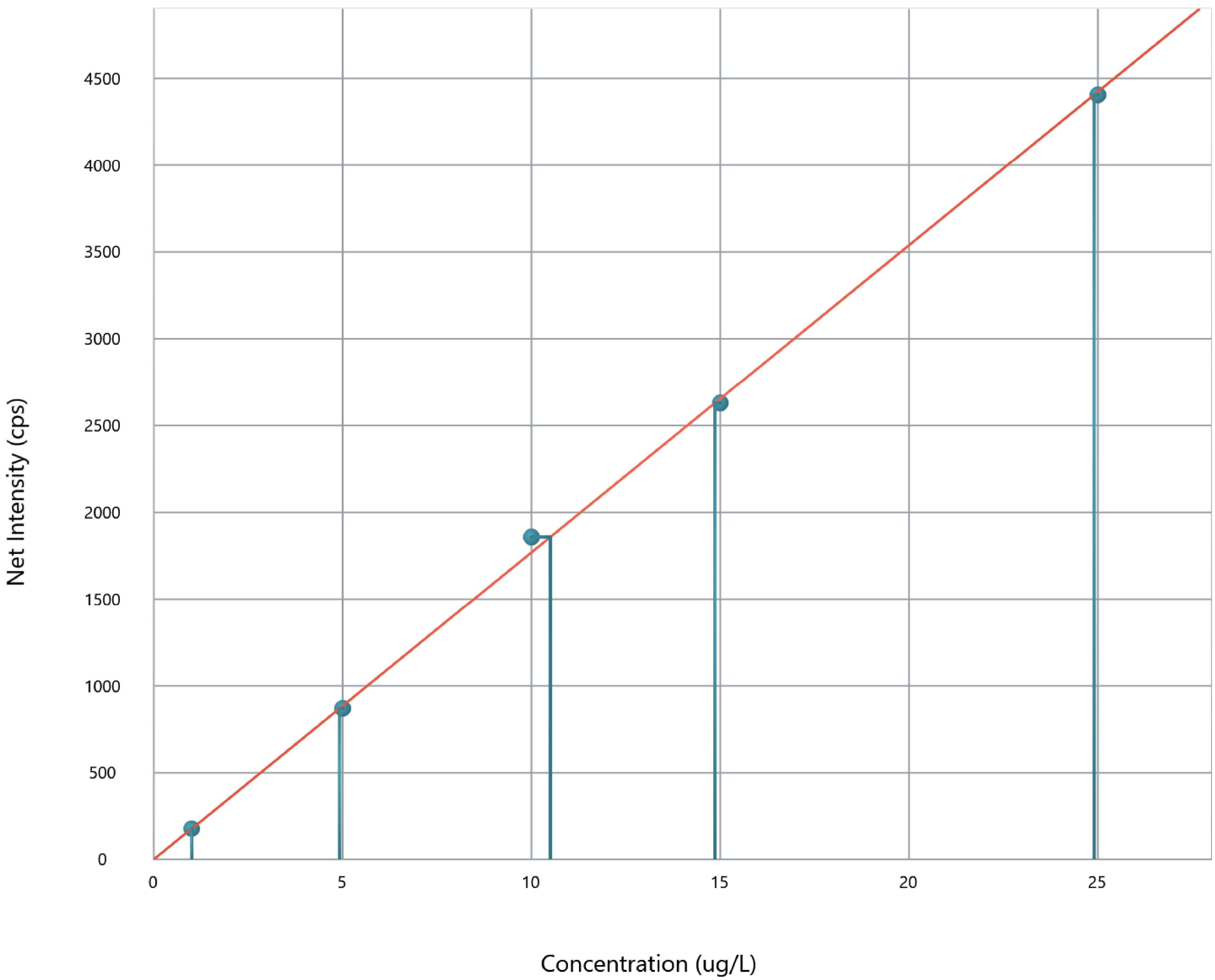

Statistics

Eqn. :	y = 177x + 0	
Cor.Coeff. :	0.999853	
BEC :	0.060285	ug/L
DL :	0.087007	ug/L

Calibration Table

	Net Intensity Cd 111 (cps)	Apparent Conc. Cd 111 (ug/L)
Blank	10.7	
Cal. Std.1	177.7	1.004
Cal. Std.2	870.4	4.919
Cal. Std.3	1858.1	10.502
Cal. Std.4	2629.2	14.860
Cal. Std.5	4405.7	24.900

Co 59 - Calibration

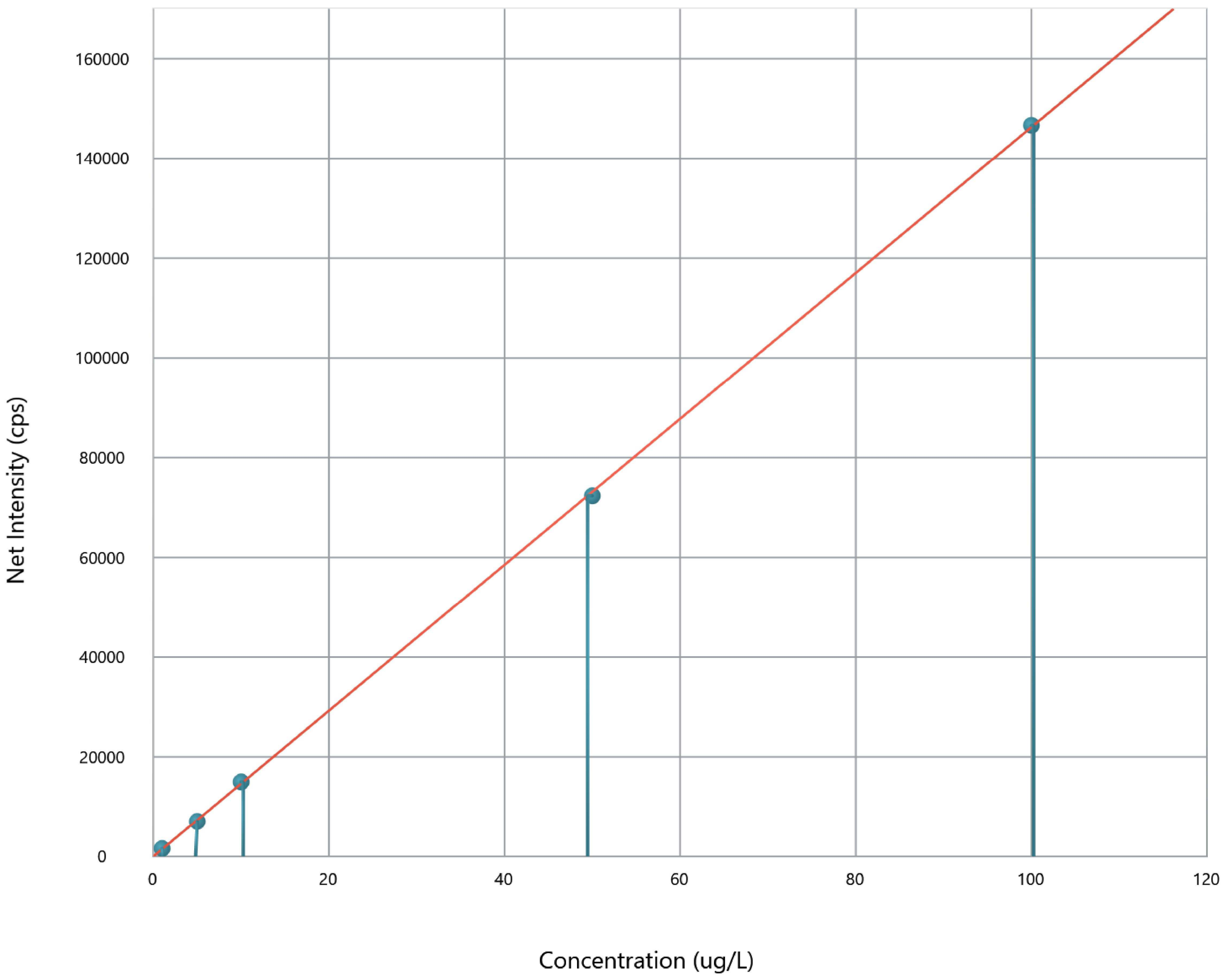

Statistics

Eqn. :	y = 1463x + 0	
Cor.Coeff. :	0.999982	
BEC :	0.018683	ug/L
DL :	0.002368	ug/L

Calibration Table

	Net Intensity Co 59 (cps)	Apparent Conc. Co 59 (ug/L)
Blank	27.3	
Cal. Std.1	1592.8	1.089
Cal. Std.2	7054.1	4.822
Cal. Std.3	14991.6	10.247
Cal. Std.4	72353.2	49.456
Cal. Std.5	146673.2	100.256

Cr 52 - Calibration

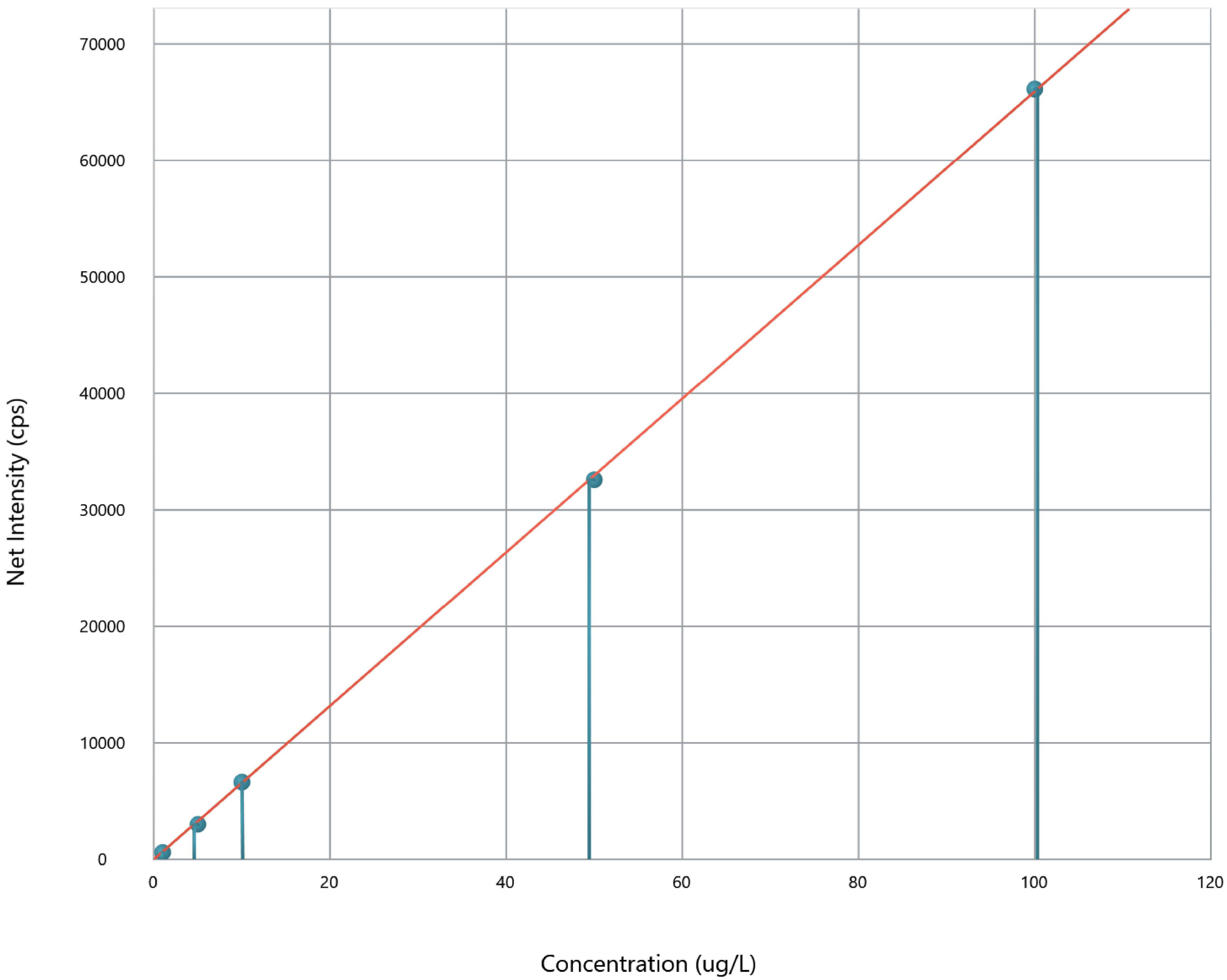

Statistics

Eqn. :	y = 659x + 0	
Cor.Coeff. :	0.999976	
BEC :	0.387793	ug/L
DL :	0.027429	ug/L

Calibration Table

	Net Intensity Cr 52 (cps)	Apparent Conc. Cr 52 (ug/L)
Blank	255.7	
Cal. Std.1	597.4	0.906
Cal. Std.2	3018.7	4.579
Cal. Std.3	6649.7	10.086
Cal. Std.4	32588.7	49.430
Cal. Std.5	66126.0	100.298

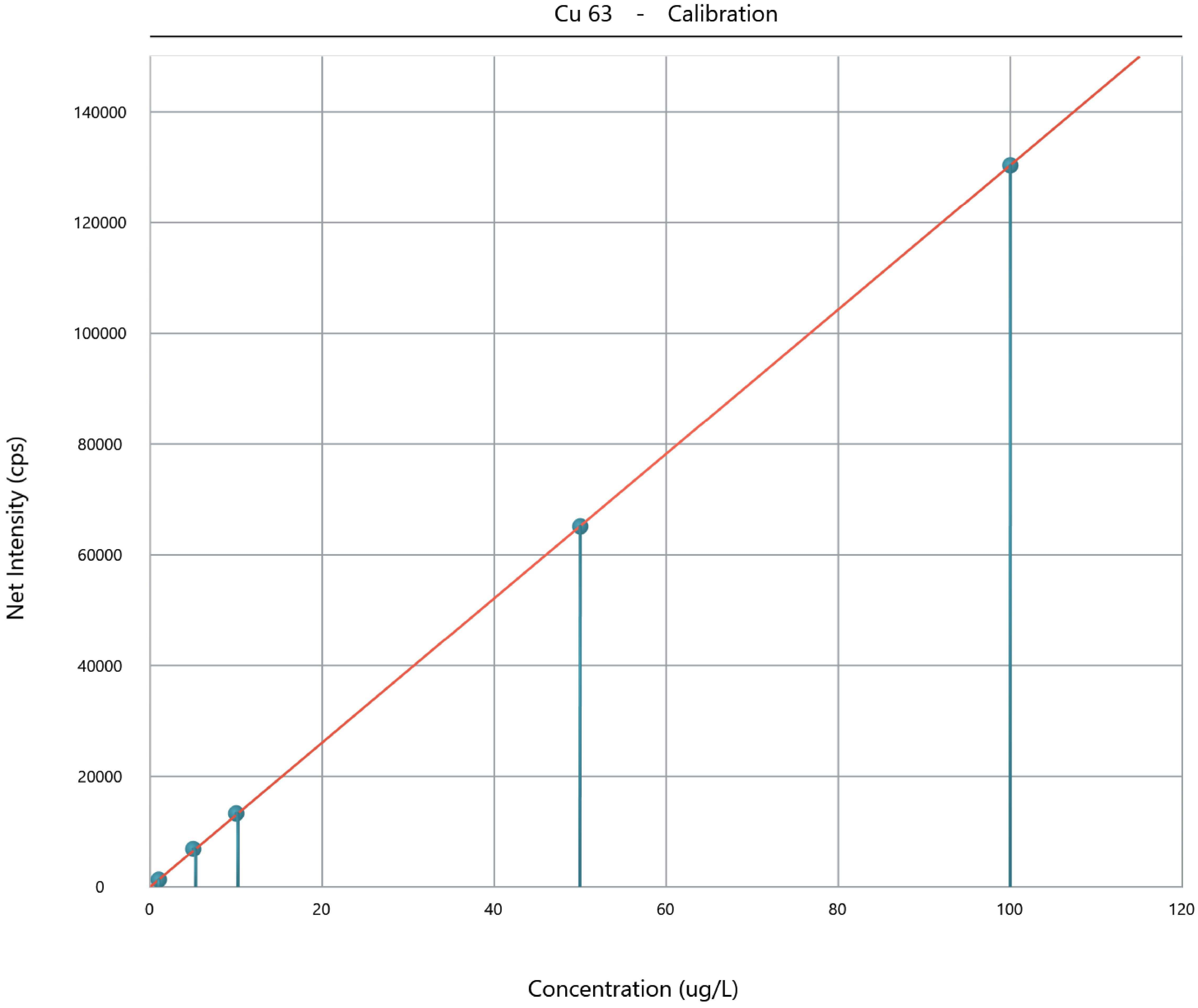

Statistics

Eqn. :	y = 1304x + 0	
Cor.Coeff. :	0.999995	
BEC :	0.255660	ug/L
DL :	0.066144	ug/L

Calibration Table

	Net Intensity Cu 63 (cps)	Apparent Conc. Cu 63 (ug/L)
Blank	333.3	
Cal. Std.1	1311.1	1.006
Cal. Std.2	6902.8	5.294
Cal. Std.3	13317.5	10.214
Cal. Std.4	65133.3	49.955
Cal. Std.5	130364.9	99.986

Fe 57 - Calibration

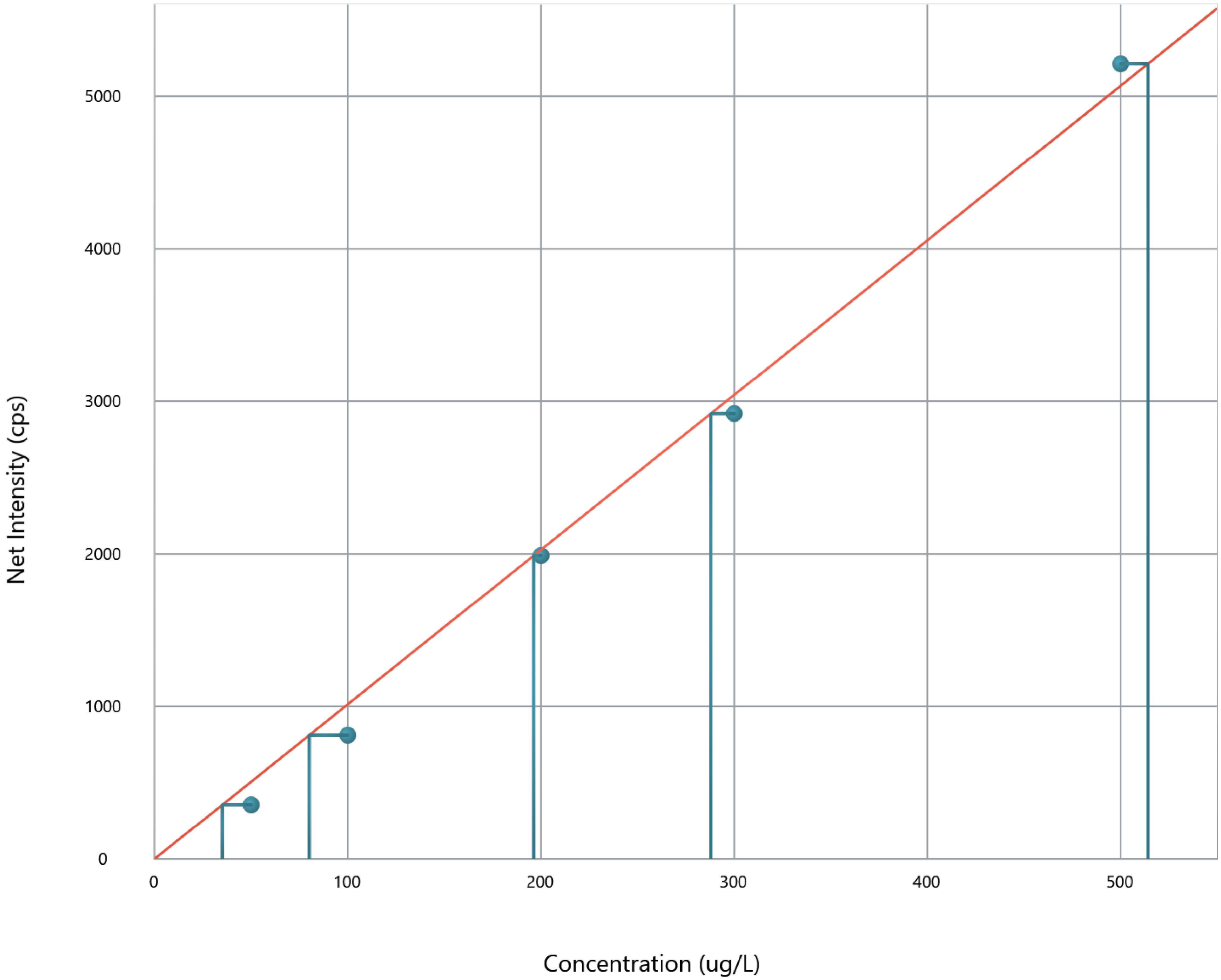

Statistics

Eqn. :	y = 10x + 0	
Cor.Coeff. :	0.998754	
BEC :	21.208174	ug/L
DL :	5.622676	ug/L

Calibration Table

	Net Intensity Fe 57 (cps)	Apparent Conc. Fe 57 (ug/L)
Blank	215.0	
Cal. Std.1	355.7	35.085
Cal. Std.2	811.7	80.068
Cal. Std.3	1990.2	196.314
Cal. Std.4	2919.0	287.937
Cal. Std.5	5212.7	514.190

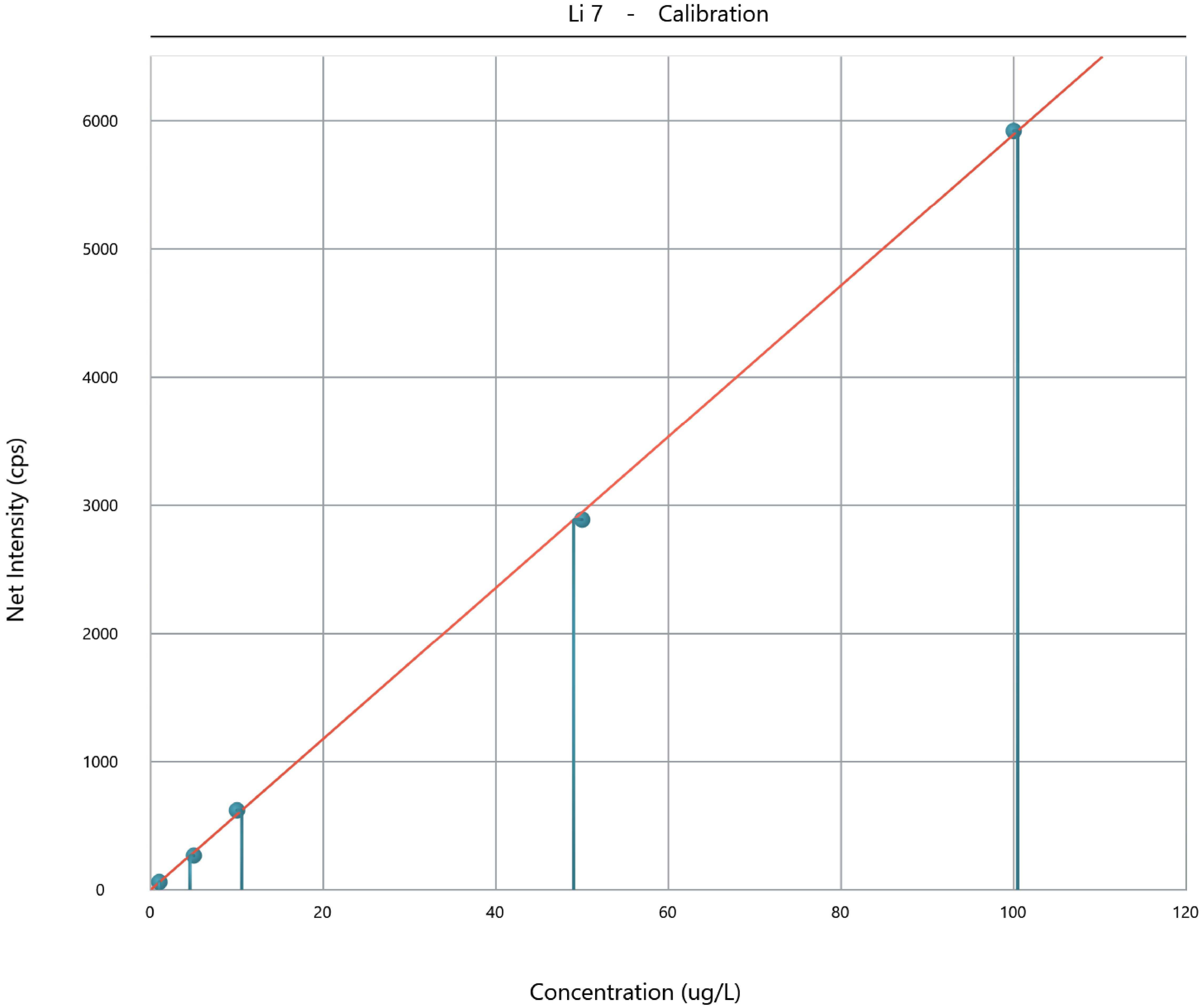

Statistics

Eqn. :	y = 59x + 0	
Cor.Coeff. :	0.999932	
BEC :	0.016967	ug/L
DL :	0.000000	ug/L

Calibration Table

	Net Intensity Li 7 (cps)	Apparent Conc. Li 7 (ug/L)
Blank	1.0	
Cal. Std.1	60.7	1.029
Cal. Std.2	268.3	4.553
Cal. Std.3	622.0	10.554
Cal. Std.4	2888.0	49.000
Cal. Std.5	5921.2	100.466

Mg 24 - Calibration

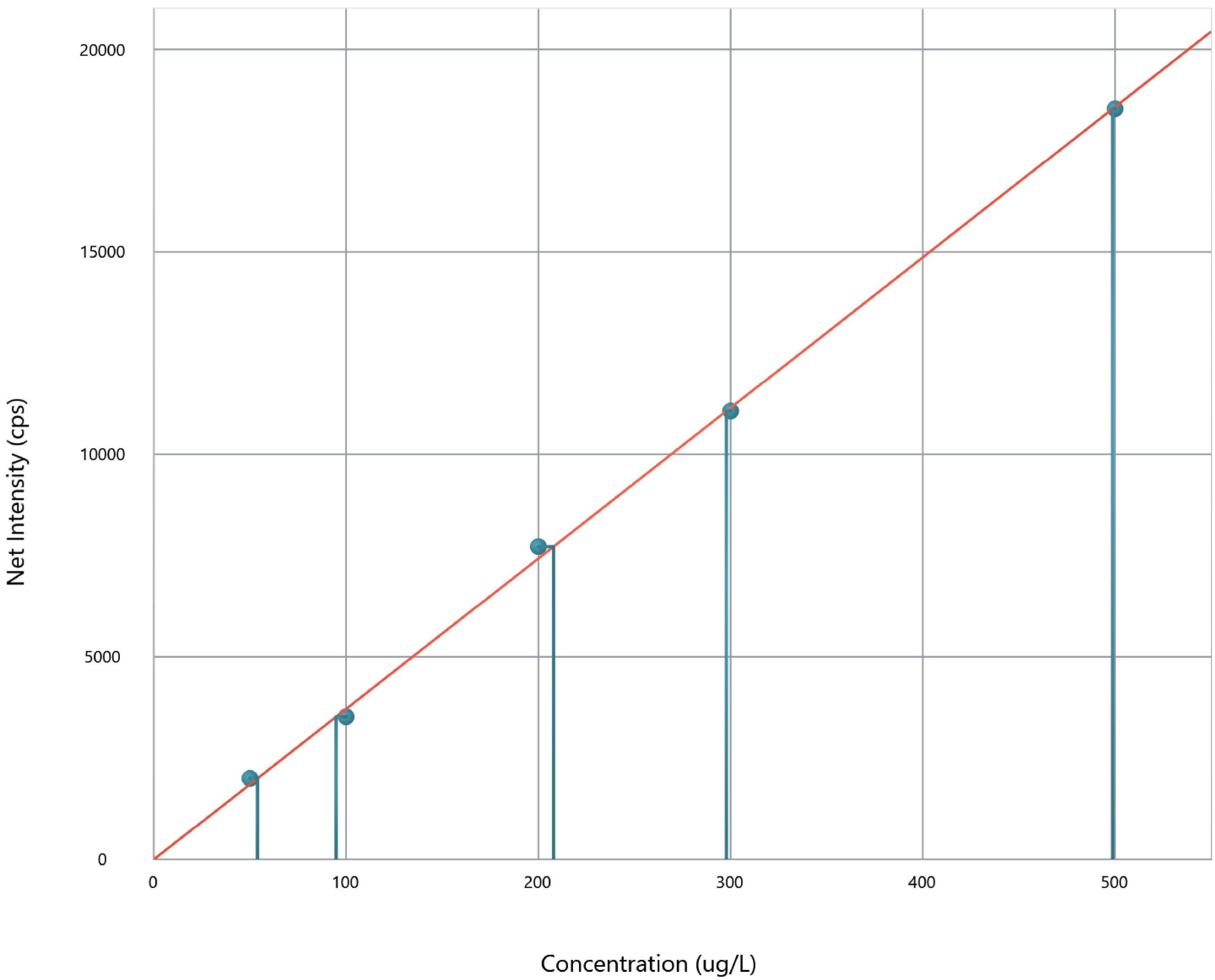

Statistics

Eqn. :	y = 37x + 0	
Cor.Coeff. :	0.999858	
BEC :	3.031434	ug/L
DL :	0.889131	ug/L

Calibration Table

	Net Intensity Mg 24 (cps)	Apparent Conc. Mg 24 (ug/L)
Blank	112.7	
Cal. Std.1	2005.2	53.951
Cal. Std.2	3524.1	94.821
Cal. Std.3	7728.2	207.934
Cal. Std.4	11069.0	297.825
Cal. Std.5	18537.5	498.772

Mn 55 - Calibration

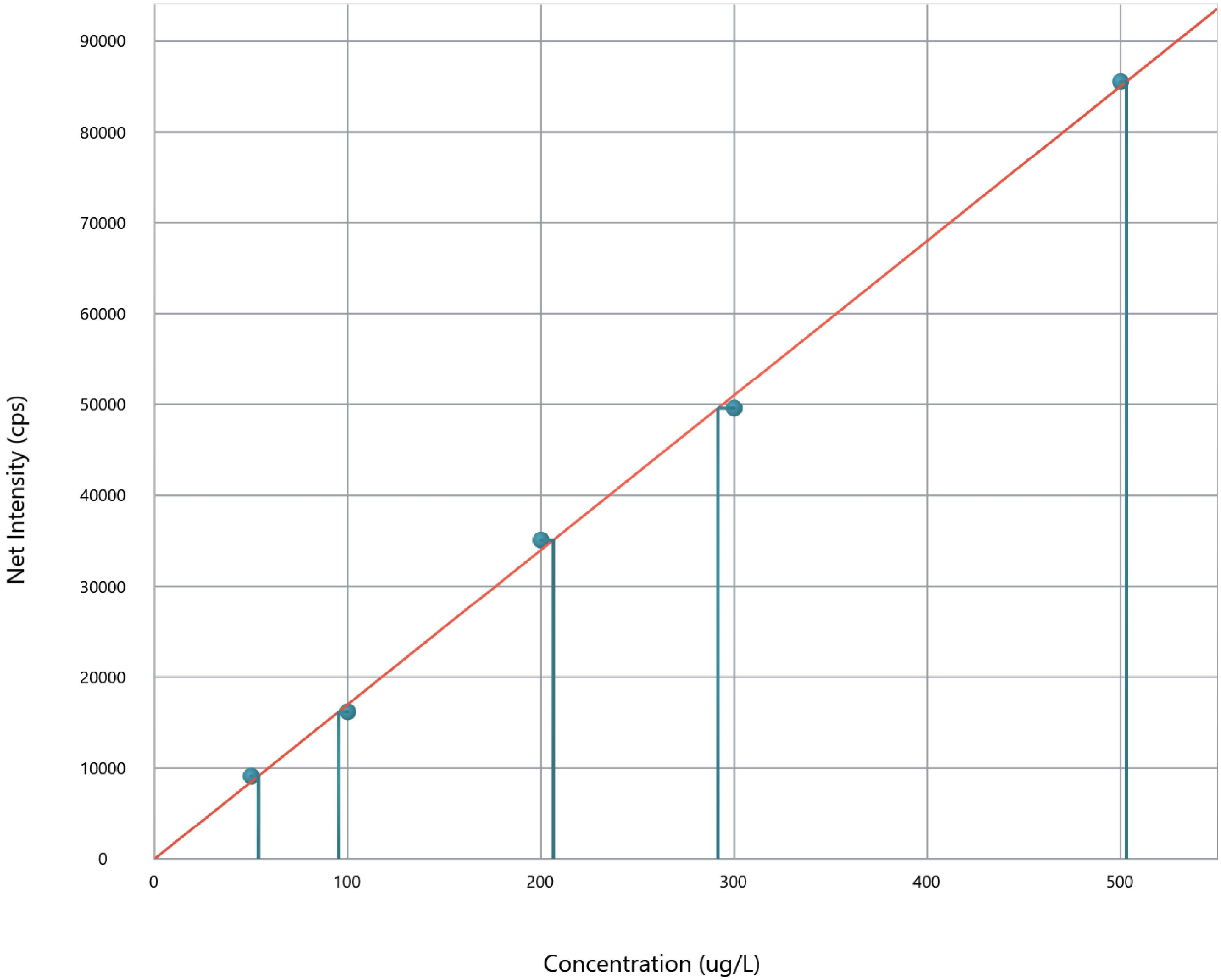

Statistics

Eqn. :	y = 170x + 0	
Cor.Coeff. :	0.999801	
BEC :	0.235213	ug/L
DL :	0.110168	ug/L

Calibration Table

	Net Intensity Mn 55 (cps)	Apparent Conc. Mn 55 (ug/L)
Blank	40.0	
Cal. Std.1	9141.3	53.754
Cal. Std.2	16199.9	95.260
Cal. Std.3	35098.8	206.392
Cal. Std.4	49596.1	291.641
Cal. Std.5	85545.0	503.031

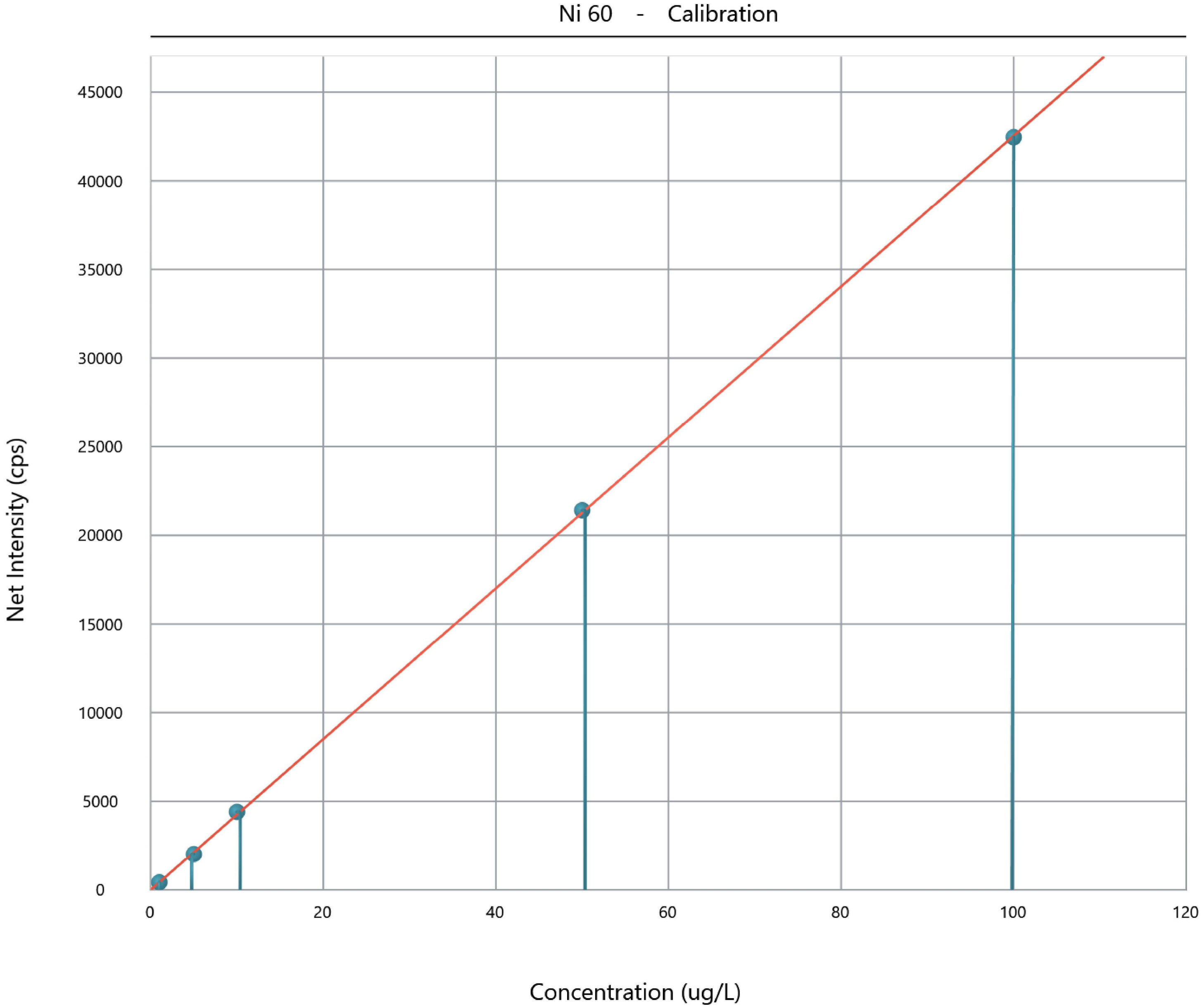

Statistics

Eqn. :	y = 425x + 0	
Cor.Coeff. :	0.999986	
BEC :	0.416049	ug/L
DL :	0.014103	ug/L

Calibration Table

	Net Intensity Ni 60 (cps)	Apparent Conc. Ni 60 (ug/L)
Blank	177.0	
Cal. Std.1	425.3	1.000
Cal. Std.2	2023.2	4.756
Cal. Std.3	4411.4	10.369
Cal. Std.4	21413.0	50.332
Cal. Std.5	42462.2	99.809

Pb 208 - Calibration

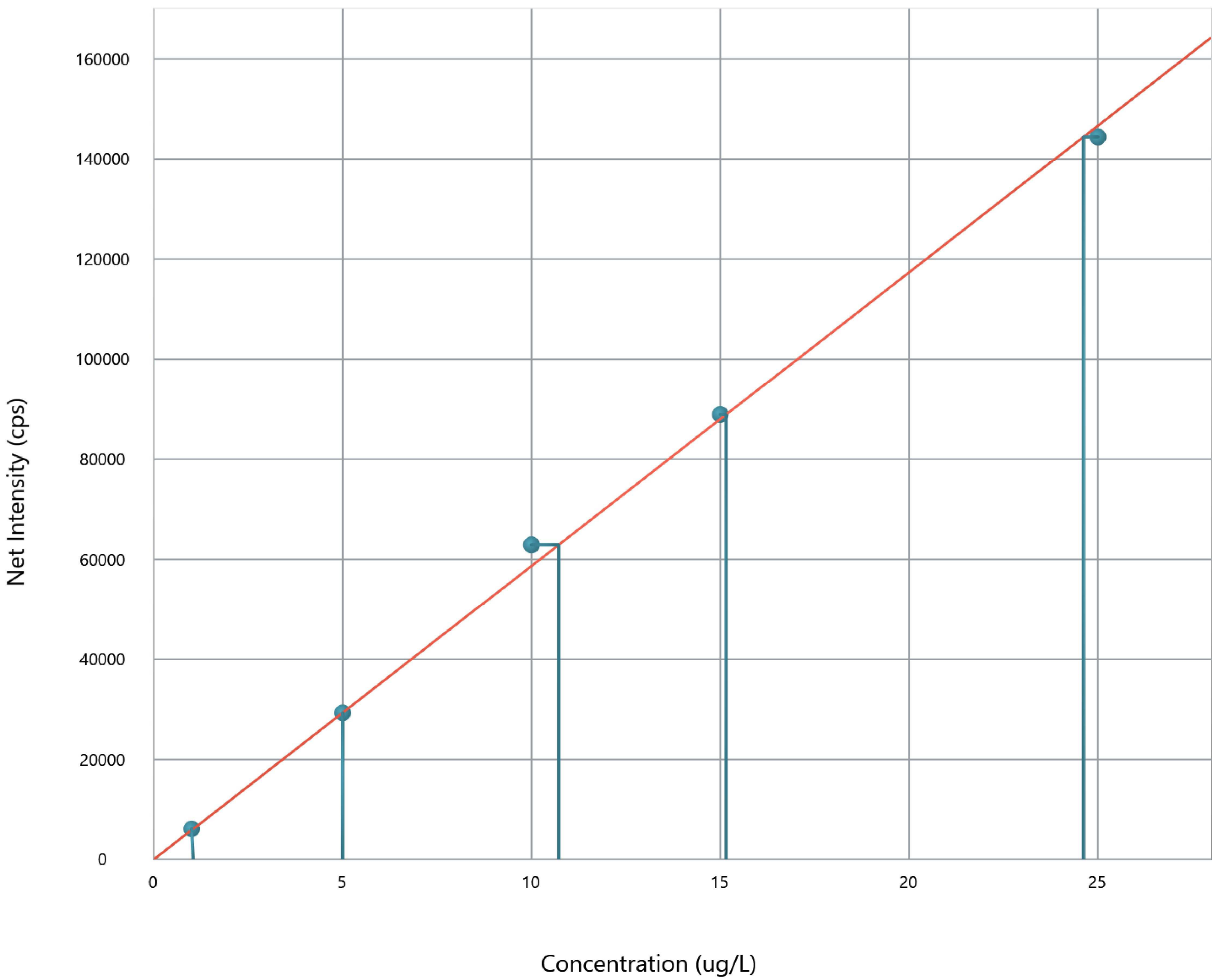

Statistics

Eqn. :	y = 5867x + 0	
Cor.Coeff. :	0.999646	
BEC :	0.228505	ug/L
DL :	0.025126	ug/L

Calibration Table

	Net Intensity Pb 208 (cps)	Apparent Conc. Pb 208 (ug/L)
Blank	1340.7	
Cal. Std.1	6110.5	1.041
Cal. Std.2	29311.1	4.996
Cal. Std.3	62907.8	10.722
Cal. Std.4	88913.2	15.154
Cal. Std.5	144445.2	24.618

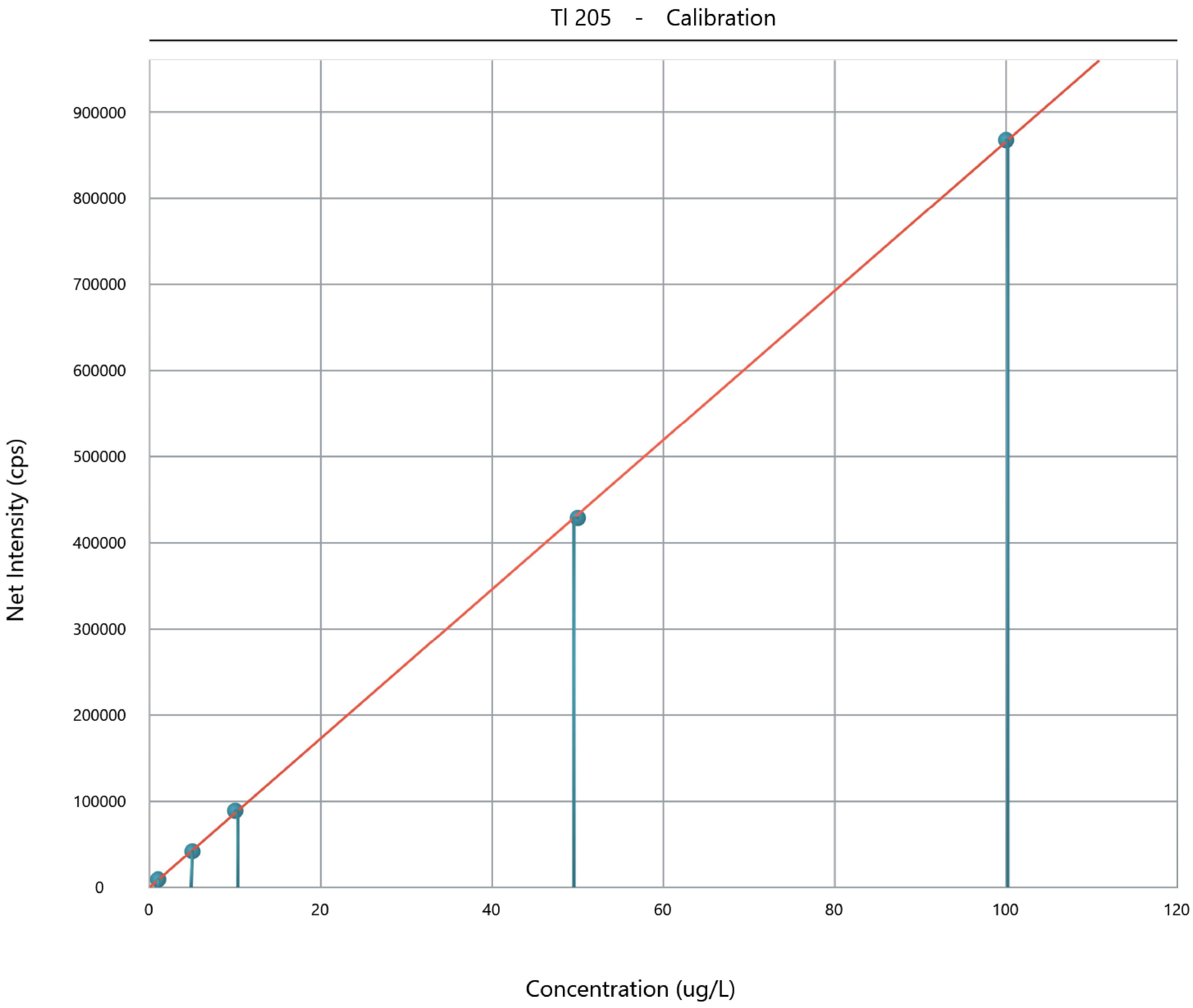

Statistics

Eqn. : y = 8658x + 0

Cor.Coeff. : 0.999985

BEC : 0.009972 ug/L

DL : 0.004061 ug/L

Calibration Table

	Net Intensity Tl 205 (cps)	Apparent Conc. Tl 205 (ug/L)
Blank	86.3	
Cal. Std.1	9201.7	1.063
Cal. Std.2	42056.1	4.858
Cal. Std.3	89242.8	10.308
Cal. Std.4	428953.4	49.546
Cal. Std.5	867533.3	100.203

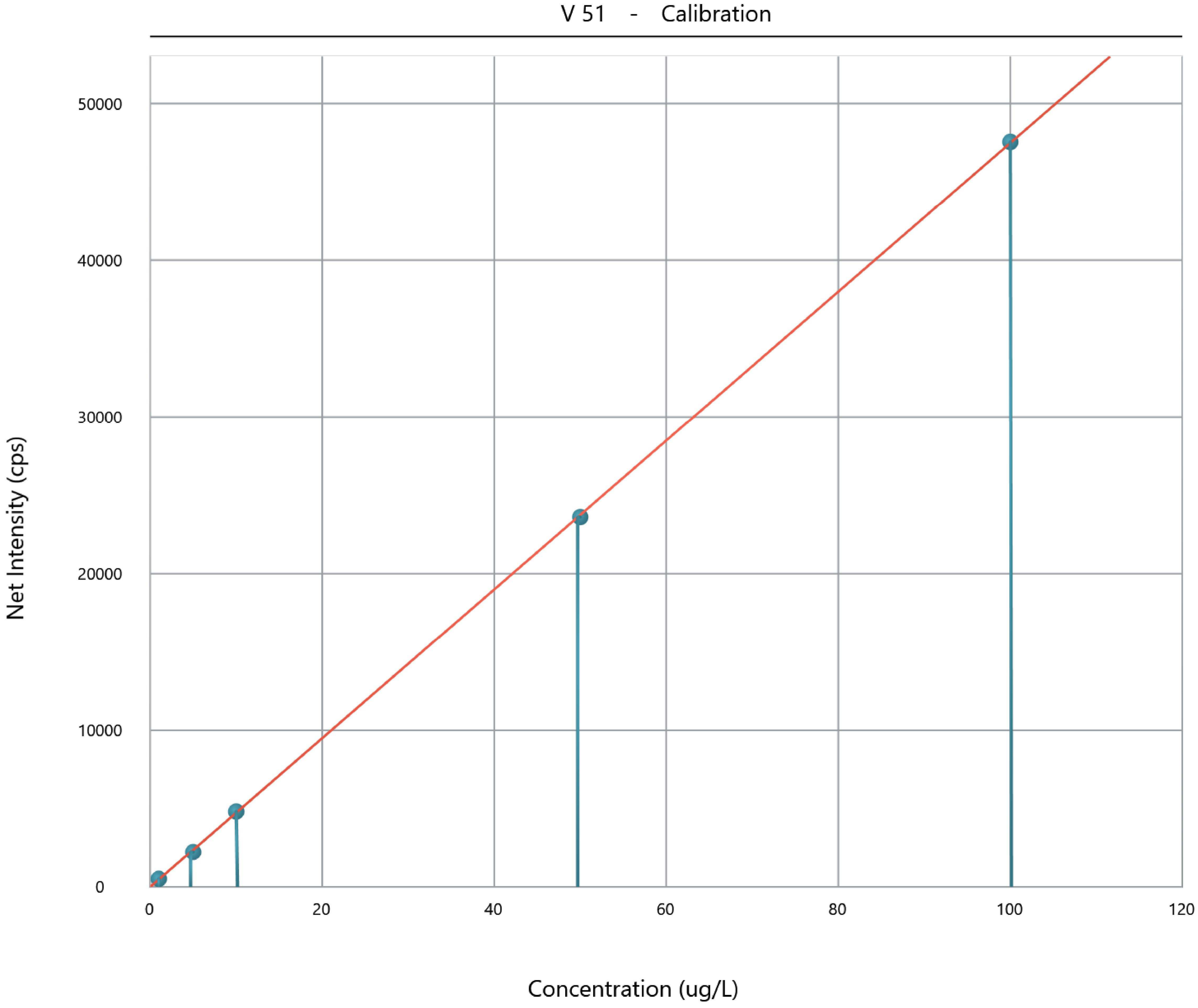

Statistics

Eqn. :	y = 475x + 0	
Cor.Coeff. :	0.999991	
BEC :	0.019654	ug/L
DL :	0.028487	ug/L

Calibration Table

	Net Intensity V 51 (cps)	Apparent Conc. V 51 (ug/L)
Blank	9.3	
Cal. Std.1	511.7	1.078
Cal. Std.2	2235.8	4.708
Cal. Std.3	4823.5	10.157
Cal. Std.4	23607.2	49.713
Cal. Std.5	47554.4	100.142

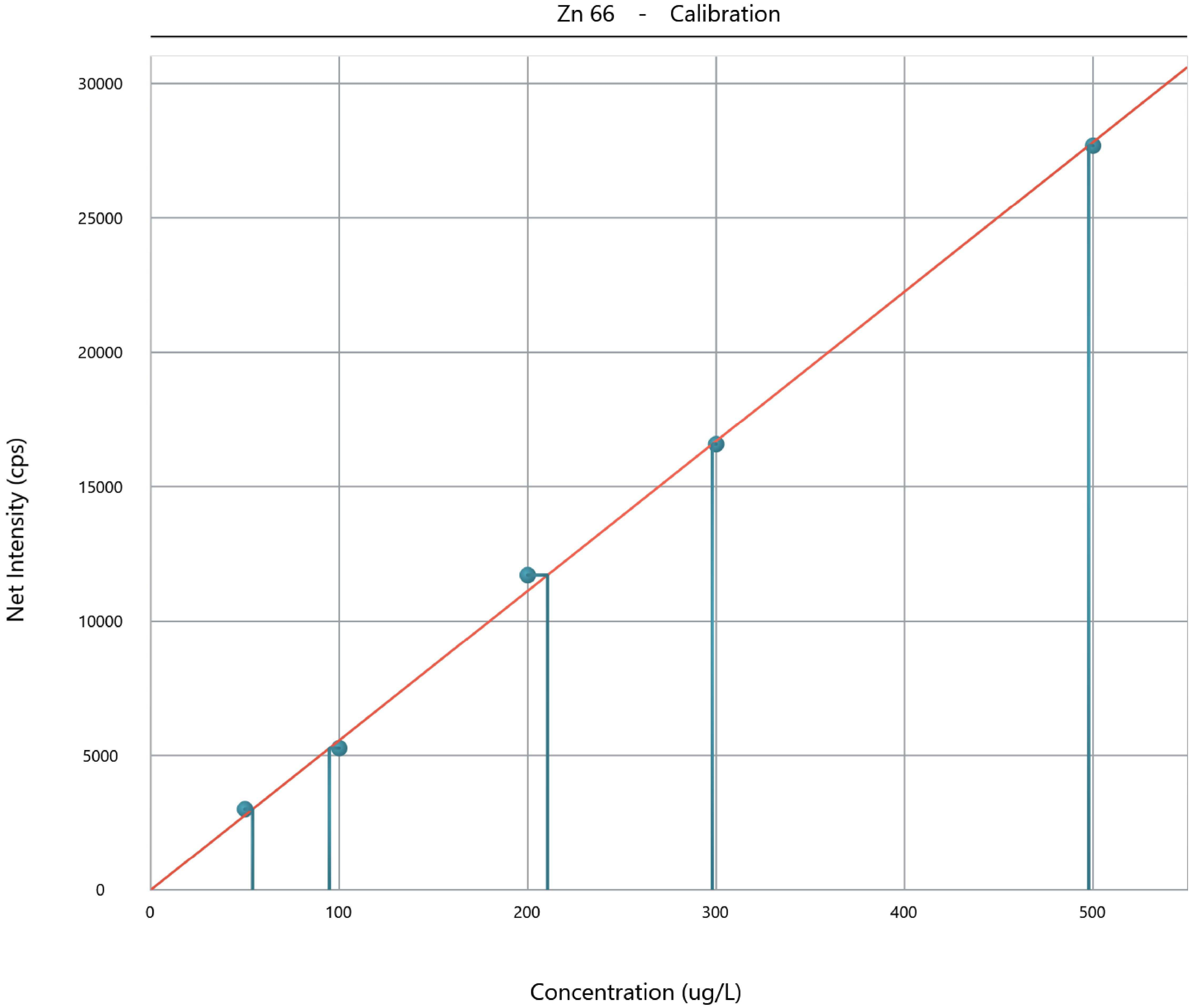

Statistics

Eqn. :	y = 56x + 0	
Cor.Coeff. :	0.999791	
BEC :	3.468381	ug/L
DL :	0.777545	ug/L

Calibration Table

	Net Intensity Zn 66 (cps)	Apparent Conc. Zn 66 (ug/L)
Blank	193.0	
Cal. Std.1	3008.4	54.062
Cal. Std.2	5274.0	94.779
Cal. Std.3	11714.6	210.521
Cal. Std.4	16579.5	297.946
Cal. Std.5	27692.9	497.662

Hg 202 - Calibration

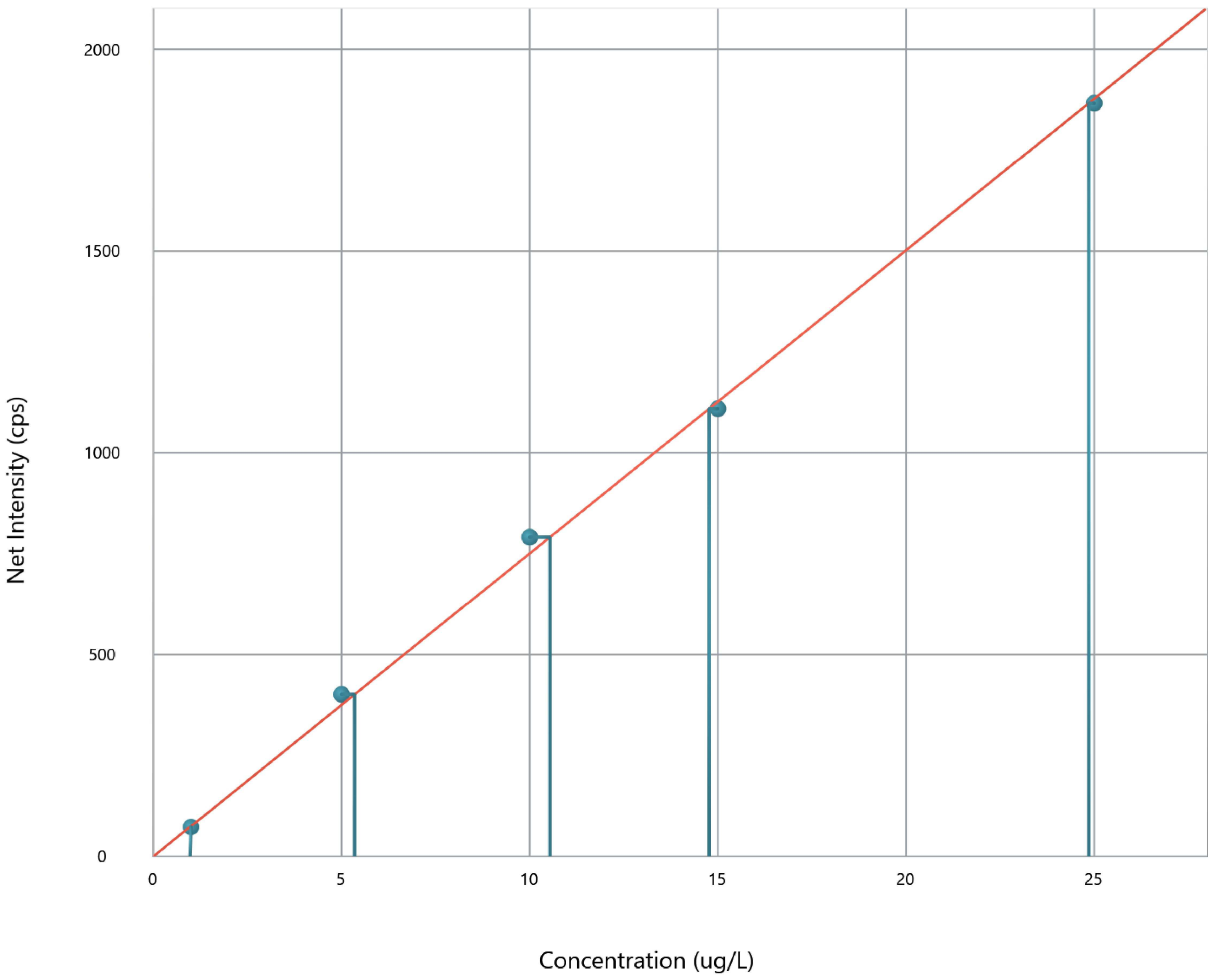

Statistics

Eqn. :	y = 75x + 0	
Cor.Coeff. :	0.999751	
BEC :	0.310685	ug/L
DL :	0.100527	ug/L

Calibration Table

	Net Intensity Hg 202 (cps)	Apparent Conc. Hg 202 (ug/L)
Blank	23.3	
Cal. Std.1	73.0	0.972
Cal. Std.2	401.7	5.348
Cal. Std.3	791.4	10.537
Cal. Std.4	1109.0	14.767
Cal. Std.5	1866.8	24.856

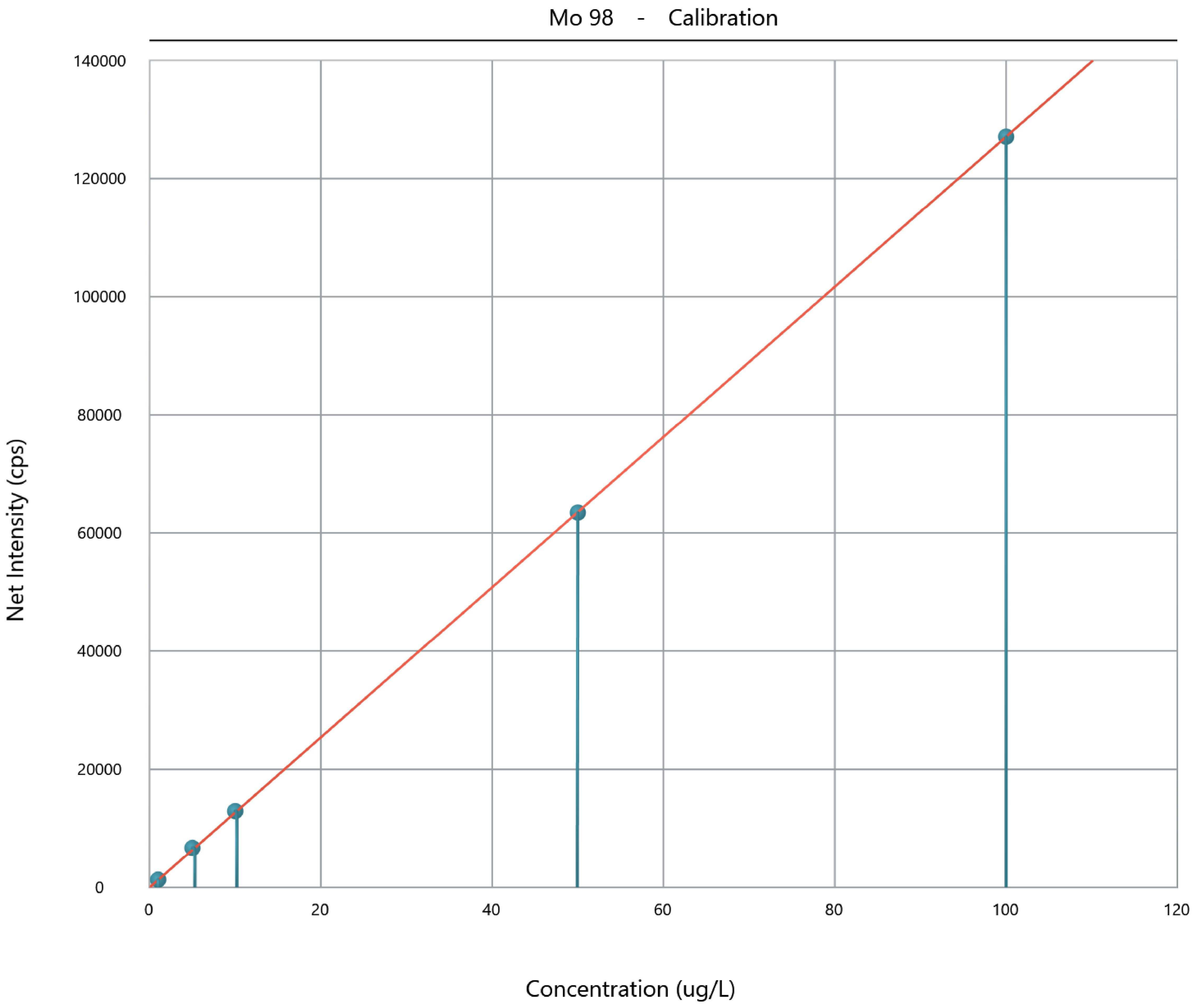

Statistics

Eqn. :	y = 1271x + 0	
Cor.Coeff. :	0.999995	
BEC :	0.132808	ug/L
DL :	0.059780	ug/L

Calibration Table

	Net Intensity Mo 98 (cps)	Apparent Conc. Mo 98 (ug/L)
Blank	168.8	
Cal. Std.1	1312.2	1.032
Cal. Std.2	6717.2	5.285
Cal. Std.3	12955.1	10.194
Cal. Std.4	63458.4	49.932
Cal. Std.5	127090.8	100.000

Pd 106 - Calibration

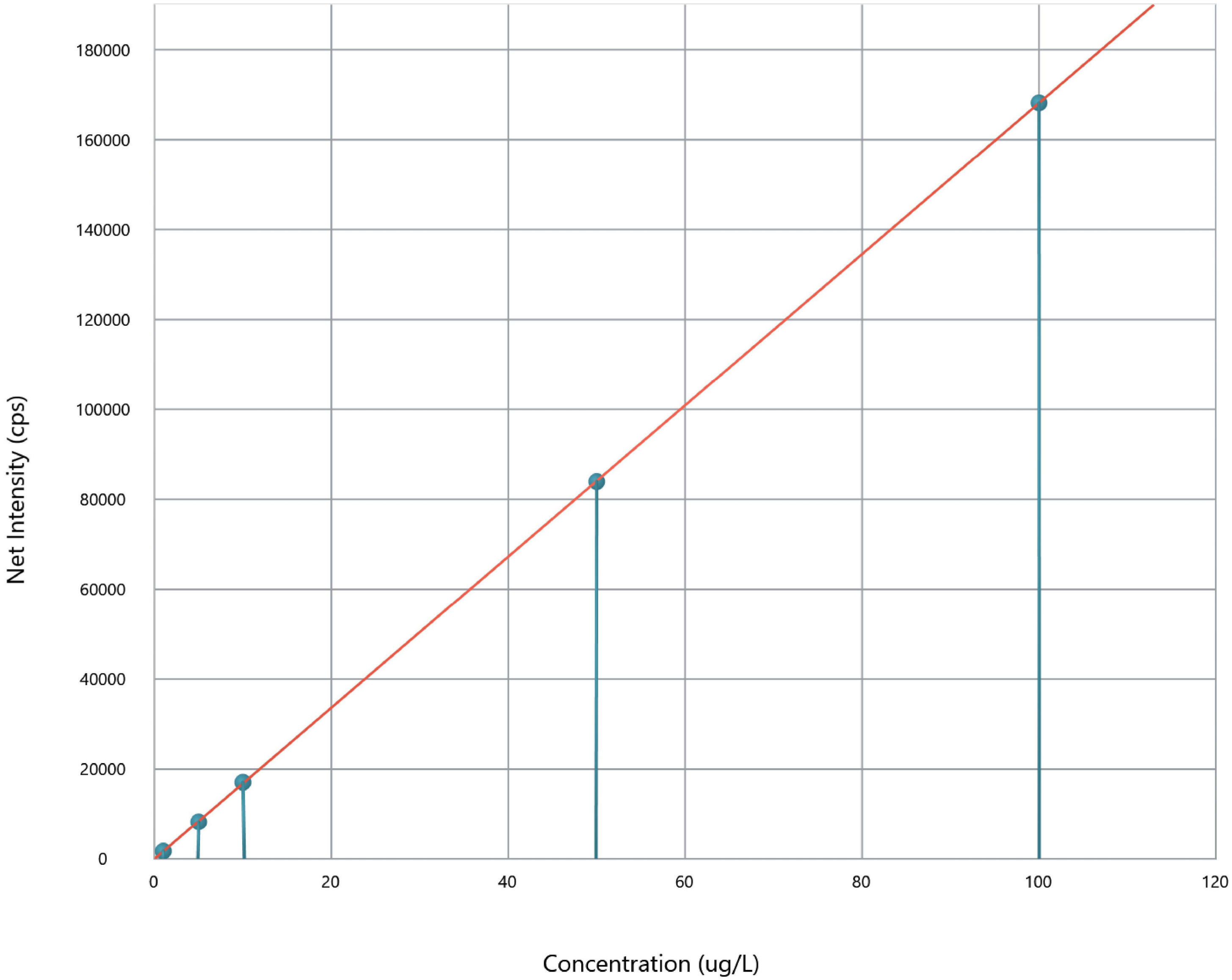

Statistics

Eqn. :	y = 1682x + 0	
Cor.Coeff. :	0.999998	
BEC :	0.071536	ug/L
DL :	0.036199	ug/L

Calibration Table

	Net Intensity Pd 106 (cps)	Apparent Conc. Pd 106 (ug/L)
Blank	120.3	
Cal. Std.1	1719.8	1.023
Cal. Std.2	8262.8	4.914
Cal. Std.3	17083.1	10.159
Cal. Std.4	83939.7	49.918
Cal. Std.5	168204.2	100.029

Pt 195 - Calibration

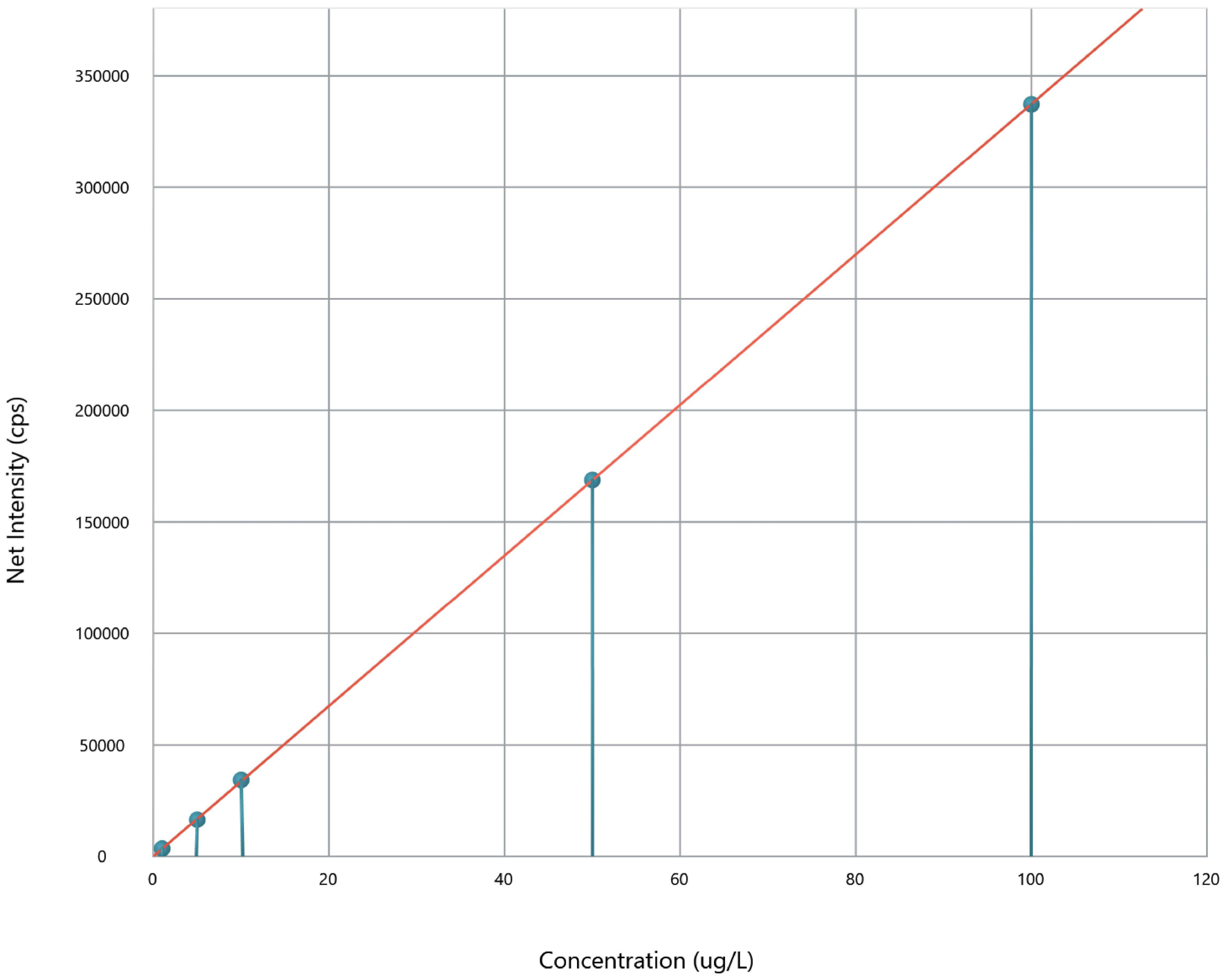

Statistics

Eqn. :	y = 3374x + 0	
Cor.Coeff. :	0.999998	
BEC :	0.002766	ug/L
DL :	0.001358	ug/L

Calibration Table

	Net Intensity Pt 195 (cps)	Apparent Conc. Pt 195 (ug/L)
Blank	9.3	
Cal. Std.1	3489.4	1.034
Cal. Std.2	16528.6	4.899
Cal. Std.3	34377.3	10.190
Cal. Std.4	168784.0	50.028
Cal. Std.5	337281.0	99.972

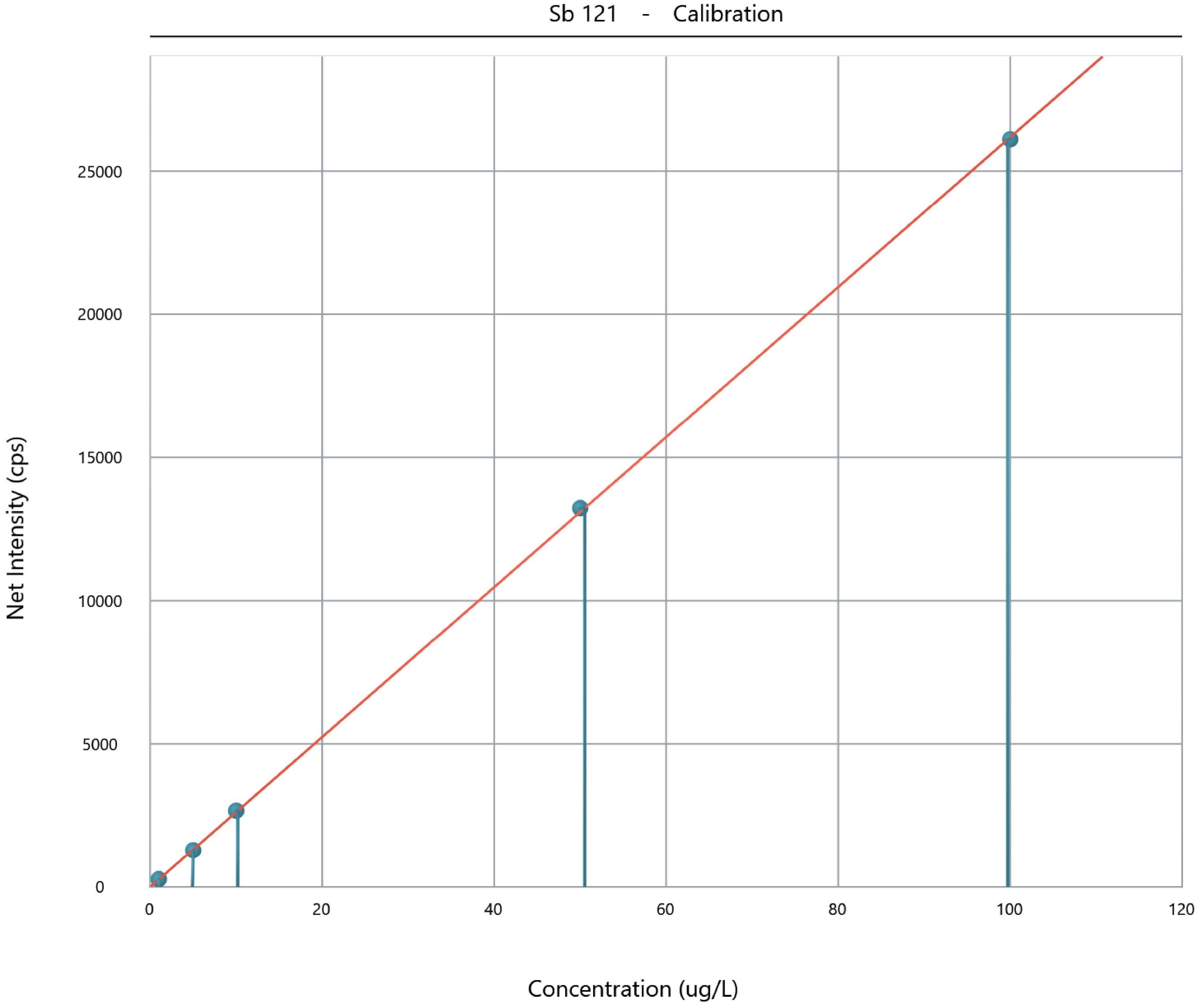

Statistics

Eqn. :	y = 262x + 0	
Cor.Coeff. :	0.999984	
BEC :	0.033100	ug/L
DL :	0.046305	ug/L

Calibration Table

	Net Intensity Sb 121 (cps)	Apparent Conc. Sb 121 (ug/L)
Blank	8.7	
Cal. Std.1	272.3	1.040
Cal. Std.2	1287.4	4.917
Cal. Std.3	2668.6	10.192
Cal. Std.4	13228.5	50.522
Cal. Std.5	26111.2	99.724
