# Supplementary material for: Elemental Analysis and In Vitro Evaluation of Antibacterial and Antifungal Activities of Usnea barbata (L.) Weber ex F.H. Wigg from Călimani Mountains, Romania
Source: Plants (Basel). 2021 Dec 23;11(1):32. doi: 10.3390/plants11010032 (PMC8747648; doi:10.3390/plants11010032)
Supplement: Supplementary file 1 [file plants-11-00032-s001.zip › Table S1. Specificity of ICP-MS elemental analysis, for two U. barbata (dUB) sample dilutions (d=10 and d=100), and (n=3) replicates; Spike Recovery % values for all 23 elements.pdf]

Table S1. Specificity of ICP-MS elemental analysis, for two *U. barbata* (dUB) sample dilutions (d=10 and d=100), and (n=3) replicates; Spike Recovery % values for all 23 elements

Sample Id	dUB d=10	dUB d=10 spiked_1	dUB d=10 spiked_2	dUB d=10 spiked_3	Average (μg/L)	Theoretical Concentration μg/L	Recovery %	dUB d=100	dUB d=100 spiked_1	dUB d=100 spiked_2	dUB d=100 spiked_3	Average (μg/L)	Recovery %
**Ag (μg/L)**	0.007	0.970	0.964	0.948	0.961	1.000	**95.388**	0.039	-0.003	-0.008	-0.010	-	**-**
**Al (μg/L)**	100.124	109.101	107.952	107.166	108.073	10.000	**79.486**	3.747	4.261	5.772	3.989	-	**-**
**As (μg/L)**	0.039	1.299	1.280	1.325	1.301	1.000	**126.231**	-0.006	0.045	-0.032	0.032	-	**-**
**Ba (μg/L)**	2.968	4.153	4.087	4.044	4.095	1.000	**112.698**	0.209	0.209	0.214	0.190	-	**-**
**Ca (μg/L)**	1576.439	1374.213	1489.114	1351.233	1404.853	50.000	**-**	128.688	197.629	151.669	216.013	188.437	**119.496**
**Cd (μg/L)**	0.126	1.336	1.287	1.179	1.268	1.000	**114.143**	0.021	0.033	0.033	0.030	-	**-**
**Co (μg/L)**	0.054	1.546	1.576	1.549	1.557	1.000	**149.392**	-0.010	-0.010	-0.008	-0.005	-	**-**
**Cr (μg/L)**	0.837	1.446	1.413	1.389	1.416	1.000	**141.600**	-0.302	-0.363	-0.346	-0.366	-	**-**
**Cu (μg/L)**	1.268	2.518	2.571	2.576	2.555	1.000	**128.659**	-0.119	-0.083	-0.074	-0.085	-	**-**
**Fe (μg/L)**	58.515	96.074	95.073	98.517	96.555	50.000	**76.080**	-12.715	26.726	26.755	26.637	-	**-**
**Li (μg/L)**	0.014	0.888	0.847	0.971	0.902	1.000	**88.848**	-0.005	-0.014	-0.014	-0.018	-	**-**

Table S1. Specificity of ICP-MS elemental analysis, for two *U. barbata* (dUB) sample dilutions (d=10 and d=100), and (n=3) replicates; Spike Recovery % values for all 23 elements

Sample Id	dUB d=10	dUB d=10 spiked_1	dUB d=10 spiked_2	dUB d=10 spiked_3	Average (µg/L)	Theoretical Concentration µg/L	Recovery %	dUB d=100	dUB d=100 spiked_1	dUB d=100 spiked_2	dUB d=100 spiked_3	Average µg/L	Recovery %
**Mg (µg/L)**	184.425	214.310	213.644	212.645	213.533	50.000	**-**	14.094	48.214	49.692	48.841	49.266	**70.345**
**Mn (µg/L)**	109.459	143.390	144.431	142.186	143.336	50.000	**-**	9.928	46.451	45.786	46.435	46.224	**72.592**
**Ni (µg/L)**	0.607	1.759	1.744	1.665	1.723	1.000	**111.593**	-0.359	-0.060	-0.070	-0.083	-	
**Pb (µg/L)**	1.351	2.150	2.129	2.085	2.122	1.000	**77.052**	-0.181	-0.087	-0.087	-0.090	-	
**Tl (µg/L)**	-0.003	0.881	0.869	0.855	0.868	1.000	**87.100**	0.061	0.016	0.017	0.016	-	
**V (µg/L)**	0.176	0.982	0.970	0.990	0.981	1.000	**80.444**	0.003	0.006	0.006	0.004	-	
**Zn (µg/L)**	25.235	85.796	85.203	82.649	84.550	50.000	**118.629**	-1.034	62.236	61.223	61.767	-	
**Hg (µg/L)**	0.677	2.010	1.831	1.942	1.928	1.000	**125.061**	-0.068	-0.013	-0.017	-0.030	-	
**Mo (µg/L)**	0.027	0.854	0.879	0.847	0.860	1.000	**83.345**	-0.005	-0.005	-0.010	-0.016	-	
**Pd (µg/L)**	0.177	1.036	1.049	1.011	1.032	1.000	**85.469**	-0.015	-0.008	-0.012	-0.011	-	
**Pt (µg/L)**	0.000	1.098	1.084	1.068	1.083	1.000	**108.305**	-0.001	-0.001	-0.001	0.000	-	
**Sb (µg/L)**	0.045	1.121	1.066	1.157	1.115	1.000	**106.999**	-0.018	0.038	0.066	0.061	-	
