# Supplementary material for: Elemental Analysis and In Vitro Evaluation of Antibacterial and Antifungal Activities of Usnea barbata (L.) Weber ex F.H. Wigg from Călimani Mountains, Romania
Source: Plants (Basel). 2021 Dec 23;11(1):32. doi: 10.3390/plants11010032 (PMC8747648; doi:10.3390/plants11010032)
Supplement: Supplementary file 1 [file plants-11-00032-s001.zip › Table S2. Determination of the detection limit value (LOD) value for each element in ICP-MS analysis of Usnea barbata dried lichen.pdf]

Table S2. Determination of the detection limit value (LOD) value for each element in ICP-MS analysis of *Usnea barbata* dried lichen

Analyte	Standard 1_1	Standard 1_2	Standard 1_3	Standard 1_4	Standard 1_5	Standard 1_6	Standard 1_7	Standard 1_8	Standard 1_9	Standard 1_10	STD DEV	SLOPE	LOD μg/L
**Ag (cps)**	3342.800	3210.700	3149.000	3219.100	3213.700	3260.400	3247.100	3122.000	3190.100	3189.400	58.085	3295.000	0.058
**Al (cps)**	92.300	103.700	98.700	100.700	106.700	116.300	110.300	123.300	121.700	111.300	9.544	11.000	2.863
**As (cps)**	47.000	46.700	41.000	51.700	58.000	41.300	51.000	56.700	49.000	50.000	5.335	51.000	0.345
**Ba (cps)**	1715.900	1703.600	1714.600	1759.300	1701.000	1681.300	1637.900	1684.300	1636.900	1706.900	34.840	1721.000	0.067
**Ca (cps)**	5.000	2.300	1.000	3.700	6.700	4.000	3.000	5.000	4.000	4.300	1.492	1.000	4.924
**Cd (cps)**	179.300	196.300	178.300	199.700	196.000	193.00	187.700	206.300	201.300	196.300	8.669	195.000	0.147
**Co (cps)**	1571.800	1584.400	1562.800	1629.100	1592.400	1598.400	1568.400	1619.800	1560.800	1524.100	29.126	1608.000	0.060
**Cr (cps)**	540.000	503.400	546.700	566.400	535.000	521.400	527.400	542.700	505.400	511.700	19.022	698.000	0.090
**Cu (cps)**	1387.100	1355.800	1369.100	1406.800	1389.400	1347.100	1349.100	1394.800	1377.800	1322.400	24.755	1441.000	0.057
**Fe (cps)**	398.011	372.677	409.679	372.677	395.678	376.677	385.344	393.345	385.011	384.678	11.308	11.000	3.392
**Li (cps)**	65.333	64.000	72.667	76.000	67.000	65.667	64.000	66.667	75.000	67.667	4.250	71.000	0.198

Table S2. Determination of the detection limit value (LOD) value for each element in ICP-MS analysis of *Usnea barbata* dried lichen

**Mg (cps)**	2103.510	2020.830	2057.170	2054.500	2104.170	2009.490	2015.830	2037.500	2039.830	2042.500	31.343	41.000	2.523
**Mn (cps)**	9224.670	8887.450	9164.960	9228.340	9134.940	9179.970	9045.220	9135.610	9062.230	8981.180	104.231	180.000	1.911
**Ni (cps)**	417.346	410.345	426.679	422.346	416.012	409.345	448.014	443.680	403.678	425.679	13.677	469.000	0.096
**Pb (cps)**	5970.750	5854.020	5723.620	5918.390	5984.090	6013.111	5703.270	5877.700	5839.010	5754.300	104.641	6477.000	0.053
**Tl (cps)**	9691.380	9549.950	9587.310	9704.050	9632.000	9666.700	9451.220	9586.310	9554.620	9624.670	72.164	9633.000	0.025
**V (cps)**	473.675	475.342	493.342	482.675	500.009	474.342	508.343	451.341	493.342	480.342	15.410	492.000	0.103
**Zn (cps)**	3401.470	3273.770	3309.110	3282.770	3359.120	3317.780	3319.440	3333.450	3374.790	3332.110	37.506	62.000	1.996
**Hg (cps)**	85.667	84.667	84.000	74.334	80.000	75.334	71.000	77.667	75.667	84.334	4.923	82.000	0.198
**Mo (cps)**	1350.590	1333.850	1309.660	1375.560	1281.290	1305.990	1279.550	1267.250	1264.100	1248.250	39.149	1332.000	0.097
**Pd (cps)**	1954.96	1919.63	1848.710	1866.300	1884.660	1864.280	1880.810	1837.640	1835.130	1865.620	35.301	1819.000	0.064
**Pt (cps)**	3682.81	3614.79	3676.470	3685.140	3697.150	3708.150	3691.48	3626.800	3668.140	3633.46	30.525	3731.000	0.027
**Sb (cps)**	277.336	268.336	286.003	301.003	278.003	290.670	296.337	262.336	274.003	281.003	11.539	275.000	0.138
